# Dental Calculus as a Tool to Study the Evolution of the Mammalian Oral Microbiome

**DOI:** 10.1093/molbev/msaa135

**Published:** 2020-05-28

**Authors:** Jaelle C Brealey, Henrique G Leitão, Tom van der Valk, Wenbo Xu, Katia Bougiouri, Love Dalén, Katerina Guschanski

**Affiliations:** m1 Department of Ecology and Genetics, Animal Ecology, Evolutionary Biology Centre, Uppsala University, Uppsala, Sweden; m2 Department of Bioinformatics and Genetics, Swedish Museum of Natural History, Stockholm, Sweden; m3 Centre for Palaeogenetics, Stockholm, Sweden

**Keywords:** metagenomics, ancient DNA, antimicrobial resistance, metagenome-assembled genomes, oral pathogens

## Abstract

Dental calculus, the calcified form of the mammalian oral microbial plaque biofilm, is a rich source of oral microbiome, host, and dietary biomolecules and is well preserved in museum and archaeological specimens. Despite its wide presence in mammals, to date, dental calculus has primarily been used to study primate microbiome evolution. We establish dental calculus as a valuable tool for the study of nonhuman host microbiome evolution, by using shotgun metagenomics to characterize the taxonomic and functional composition of the oral microbiome in species as diverse as gorillas, bears, and reindeer. We detect oral pathogens in individuals with evidence of oral disease, assemble near-complete bacterial genomes from historical specimens, characterize antibiotic resistance genes, reconstruct components of the host diet, and recover host genetic profiles. Our work demonstrates that metagenomic analyses of dental calculus can be performed on a diverse range of mammalian species, which will allow the study of oral microbiome and pathogen evolution from a comparative perspective. As dental calculus is readily preserved through time, it can also facilitate the quantification of the impact of anthropogenic changes on wildlife and the environment.

## Introduction

Dental plaque is the microbial biofilm that forms on mammalian teeth (Jin and Yip 2002). Throughout an individual’s life, dental plaque undergoes periodic, sequential mineralization to form dental calculus (Jin and Yip 2002). The calcified matrix of dental calculus preserves biomolecules like DNA and proteins, which are protected from invasion of external microorganisms following the host’s death and thus preserve the authentic oral microbiome (Adler et al. 2013; Warinner et al. 2014). Dental calculus is readily available from museum-preserved and archaeological specimens and is easily sampled without damaging the underlying tooth morphology. Archaeological dental calculus has been shown to be a rich source of information on the oral microbial community, potential pathogens, host DNA, and dietary components (Armitage 1975; Dobney and Brothwell 1988; Adler et al. 2013; de la Fuente et al. 2013; Warinner et al. 2014). Dental calculus thus provides the opportunity to study oral microbiome evolution through time and to integrate investigations of microbial, dietary, and host genetic factors from the same source material (Adler et al. 2013; Warinner et al. 2014; Weyrich et al. 2017; Mann et al. 2018; Ottoni et al. 2019; Ozga et al. 2019; Modi et al. 2020).

To date, archaeological dental calculus has been primarily studied in humans, where DNA sequencing has revealed shifts in oral microbiome composition associated with cultural transitions and allowed tracking of host–pathogen coevolution (Adler et al. 2013; Warinner et al. 2014; Weyrich et al. 2017). Many other mammals produce dental calculus (e.g., Dobney and Brothwell 1988), but so far the study of nonhuman oral microbiomes from the past has received little attention. Although natural history collections have been extensively used for population genomics studies and provided insight into temporal changes within animal populations (van’t Hof et al. 2011; Holmes et al. 2016; van der Valk et al. 2018; van der Valk, Díez-del-Molino, et al. 2019), they remain virtually unexplored for the study of nonhuman oral microbiome evolution.

To establish dental calculus as a standard research tool for the study of host-associated microbiome evolution in diverse mammalian species, we used DNA sequencing to characterize the historical dental calculus microbiome of three evolutionarily distant mammalian species with distinct ecology, diet, and physiology: European reindeer (*Rangifer tarandus*), Scandinavian brown bear (*Ursus arctos*), and eastern gorilla (*Gorilla beringei*). Reindeer are group-living ruminant herbivores with a multigastric digestive system and specialized hypsodont molars adapted to an abrasive, fibrous diet. Brown bears are solitary omnivores with brachydont molars more adapted for a partially carnivorous diet. Gorillas are group-living folivores and specialized hindgut fermenters. The close evolutionary relationship between gorillas and humans (the major source of the microbial reference databases used for microbiome taxonomic characterization) and the previous successful reconstruction of the chimpanzee oral microbiome from dental calculus (Weyrich et al. 2017; Ozga et al. 2019) prompted us to include gorillas to aid characterization of previously unexplored microbiomes from the other two host species. We outline strategies to overcome the challenges of working with historical microbial DNA from nonmodel host species, including postmortem contamination and reference database biases, and demonstrate that a wealth of evolutionary, ecological, and conservation-relevant information can be obtained from historical dental calculus samples of diverse host species.

## Results

### Sample Processing and Data Authentication

**Fig. 1.**
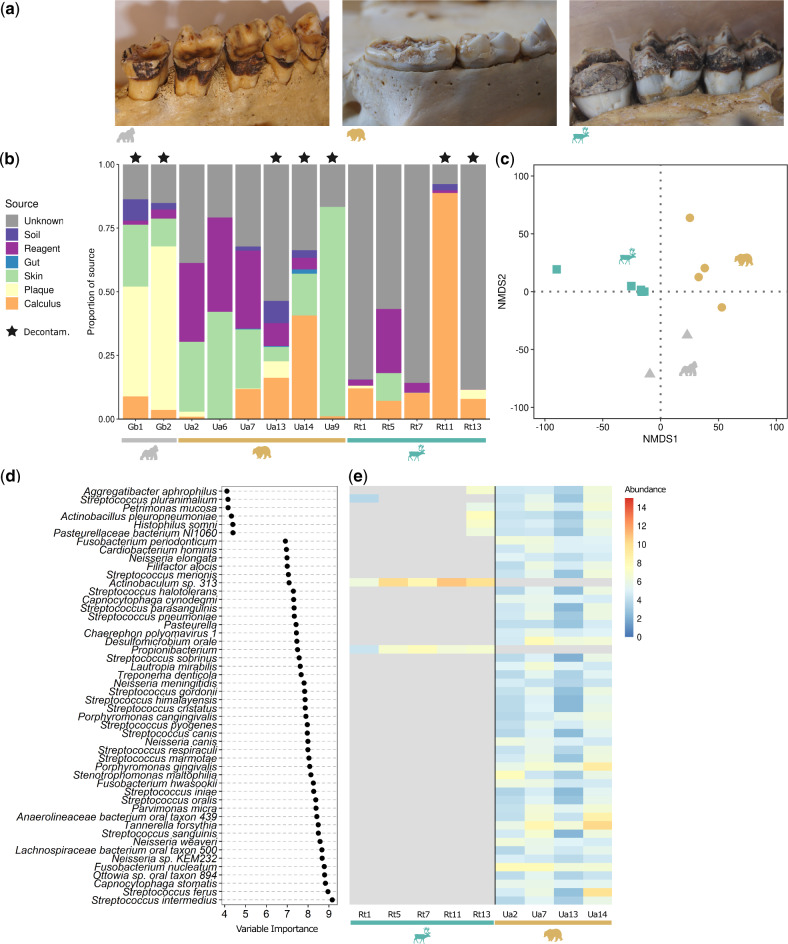
Dental calculus of nonhuman mammals shows an oral microbiome signature and contains host-specific taxa. (*a*) Dental calculus on the healthy teeth of a gorilla (*Gorilla beringei*), brown bear (*Ursus arctos*), and reindeer (*Rangifer tarandus*) specimen (left-to-right, respectively). Note these are representative photos of specimens in the museum collections. (*b*) Proportions of source contributions to the microbial communities (identified taxonomically at the species and genus level) of the dental calculus samples estimated by SourceTracker. Stars above bars indicate samples in which surface decontamination (UV and/or EDTA wash) was performed before DNA extraction. (*c*) NMDS ordination on Euclidean distance matrix of CLR normalized microbial abundances of taxa in samples from healthy teeth, colored by host species. NMDS stress: 0.107. (*d*) Random forest variable importance plot of the 30 most discriminatory taxa comparing bear and reindeer samples from healthy teeth, based on presence/absence of microbial taxa after contamination filtering. (*e*) CLR normalized abundance of the top 30 taxa in (*d*) in the bear and reindeer samples from healthy teeth. Taxa that were not detected in a sample are colored gray. Sample Ua9 from a carious bear was excluded in the analyses for (*c*–*e*), see supplementary figure S7, Supplementary Material online, for analysis with Ua9.

**Fig. 2.**
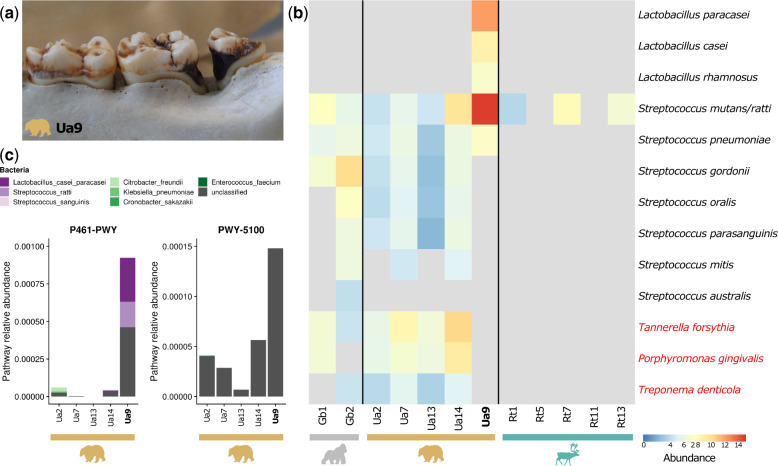
Identification of opportunistic oral pathogens in a specimen with evidence of oral disease. (*a*) Sampling site from a caries lesion from the bear specimen Ua9. (*b*) Kraken2 CLR normalized abundance of potentially cariogenic bacteria (*Streptococcus* and *Lactobacillus* species) and periodontal pathogens (*Treponema denticola*, *Porphyromonas gingivalis*, and *Tannerella forsythia*, highlighted in red text). Taxa that were not detected in a sample are colored gray. The caries bear Ua9 is highlighted in bold text. (*c*) Relative abundance of MetaCyc metabolic pathways involved in sugar fermentation to acids in brown bear samples (P461-PWY: hexitol fermentation to lactate, formate, ethanol, and acetate; PWY-5100: pyruvate fermentation to acetate and lactate). Relative contribution of bacterial species to each pathway in each sample is shown, where known. Note the differences in *y* axis scale for each pathway.

DNA extraction, Illumina shotgun sequencing, and metagenomics analyses were carried out on dental calculus dating from 1861 to 1961 collected from five reindeer (including forest, mountain, and high arctic Svalbard ecotypes), six brown bears (from western and eastern Europe), and two eastern gorilla subspecies (one Grauer’s and one mountain gorilla) (supplementary table S1, Supplementary Material online). Calculus was sampled from healthy teeth (fig. 1*a*) in all except one brown bear specimen (Ua9), which was sampled from a caries lesion (fig. 2*a*). Dental calculus on healthy teeth differed in appearance between the three host species (fig. 1*a*) and lacked the three-dimensional structure commonly observed in humans (Warinner et al. 2014). To confirm that the observed material was representative of the calcified dental plaque microbial biofilm, rather than microbes associated with the tooth surface alone, we also sequenced DNA from a segment of historical reindeer tooth, free of surface calculus. Following sequencing, microbial taxonomic assignment with Kraken2 (Wood and Salzberg 2014), and data authentication (supplementary fig. S1, Supplementary Material online, also see below), the reindeer tooth sample showed a distinct microbial community, clustering separately from calculus samples of all studied host species (supplementary fig. S2, Supplementary Material online). Microbial source analysis with the Bayesian classification tool SourceTracker (Knights et al. 2011) demonstrated that the majority of the calculus samples included microbial taxa found in human dental plaque and calculus microbial communities, whereas the microbial community associated with the tooth sample was most similar to human skin, laboratory reagent, and soil microbial communities (supplementary fig. S3, Supplementary Material online).

Contamination of samples with modern DNA is a major problem faced by all historical genomic and metagenomic studies (Key et al. 2017). We therefore used a combination of laboratory and bioinformatics procedures to reduce and quantify contamination. To control for background laboratory contamination, two blank negative controls (one during DNA extraction and one during library preparation) were taken through the entire laboratory, sequencing, and data analysis process. In the laboratory, we tested surface decontamination treatments on the two gorilla dental calculus samples (supplementary fig. S3, Supplementary Material online). An ethylenediaminetetraacetic acid (EDTA) wash was found to result in the highest proportions of oral microbes (supplementary fig. S3, Supplementary Material online), thus only the two gorilla samples with this treatment were used for subsequent analyses. For reindeer and bear calculus samples, we used a combined surface decontamination procedure of UV exposure followed by an EDTA wash (Ozga et al. 2016). However, a subset of samples was processed without any surface decontamination (supplementary fig. S3 and table S1, Supplementary Material online). Microbes associated with soil and human skin were the most common contamination sources in the negative controls (supplementary fig. S3, Supplementary Material online). Human skin and laboratory reagent taxa were particularly abundant in bear and reindeer specimens that did not undergo surface decontamination before DNA extraction (supplementary fig. S4, Supplementary Material online). Irrespective of decontamination, nonmetrical multidimensional scaling (NMDS) ordination based on microbial taxa abundances clearly separated all calculus samples, negative controls, and the reindeer tooth sample (supplementary fig. S2 and table S2, Supplementary Material online).

We employed a multistep bioinformatics approach to identify and filter out contaminant taxa that were not removed during the laboratory procedures (supplementary fig. S1, Supplementary Material online). We flagged taxa as contaminants if they were present in the negative controls (Salter et al. 2014), had higher relative abundance in low biomass samples (Davis et al. 2018), or had predominantly long DNA fragments (supplementary fig. S5, Supplementary Material online). This procedure reduced the proportion of contaminants and systematically increased the proportion of the bacterial communities attributed to the oral microbiome in our samples (supplementary fig. S3, Supplementary Material online). However, one bear sample (Ua6) contained high levels of contaminants (>70%) and no detectable oral microbiome signature after contamination filtering and was therefore excluded from all microbial analyses.

### Oral Microbiome Signature Can Be Successfully Recovered from Dental Calculus of Nonhuman Mammals

Dental calculus samples from all three host species showed a clear oral microbiome signature, although the proportion of putatively oral taxa varied substantially by sample (fig. 1*b* and supplementary fig. S6, Supplementary Material online). Each host species had a distinct oral microbiome composition (fig. 1*c*) and ∼37% of the variation between samples could be explained by host species (*F*(2,5) = 2.620, *P *<* *0.001), supplementary table S2, Supplementary Material online). Surface decontamination (performed/not performed), sequencing depth, and abundance of human reads as measure of human DNA contamination each explained <10% of the variation (supplementary table S2, Supplementary Material online). Microbial taxa that were unique to each host species were generally associated with either the mammalian oral microbiome or other mammalian body sites (supplementary table S3, Supplementary Material online). Several microbial taxa specific to the reindeer were identified as related to rumen-associated *Methanobrevibacter* species of bovines and ovines, including *M. ruminantium* and *M. olleyae* (Janssen and Kirs 2008). To independently verify the host species-specific differences between reindeer and bear (gorillas were excluded due to the low sample size of 2), we used a random forests classifier on presence/absence data from healthy teeth (for analyses including the caries bear sample, see supplementary fig. S7, Supplementary Material online). The correct host species could be assigned in all cases, and the most important taxa for determining the host species were generally related to oral bacteria, such as *Streptococcus* species, which were highly abundant in the bears but almost entirely absent in reindeer (fig. 1*d* and *e*). Reindeer also had lower microbial diversity than bears and gorillas (supplementary fig. S8, Supplementary Material online, Shannon diversity index median ± interquartile range: 1.777 ± 0.288 [reindeer] vs. 3.794 ± 0.014 [gorilla] vs. 3.836 ± 0.602 [bear], *P *=* *0.003).

Since our brown bear specimens included one sampled from a caries lesion (Ua9, fig. 2*a*), we also investigated whether taxa related to human opportunistic oral pathogens could be recovered from the dental calculus of wild animals. The most abundant bacteria in Ua9 included taxa related to members of the *Lactobacillus casei* group (*L. casei*, *L. paracasei*, *L. rhamnosus*, and *L. zeae*) and mutans streptococci (such as the closely related *Streptococcus mutans* and *S. ratti* [Gao et al. 2014])—species that have been associated with caries lesions in humans (Tanzer et al. 2001; Neves et al. 2017) (fig. 2*b*). In contrast, samples from bears, gorillas, and reindeer without signs of caries had lower relative abundances of mutans streptococci and lacked members of the *L. casei* group. One bear individual had signs of caries (Ua7) but was sampled from a healthy tooth, rather than a caries lesion, and its microbial community appeared more similar to the other bears without signs of dental caries (fig. 2*b*). The periodontal “red complex” pathogens (*Porphyromonas gingivalis*, *Treponema denticola*, and *Tannerella forsythia*) (Socransky et al. 1998) were identified in most bear and gorilla samples (fig. 2*b*).

### Functional Repertoire of the Mammalian Oral Microbiome

By performing shotgun sequencing, we were able to gain first insights into the functional potential of the microbial communities captured in dental calculus from wild animals. We used the HUMAnN2 pipeline (Franzosa et al. 2018) to assign KEGG orthologs (Kanehisa and Goto 2000) to the filtered microbial reads, and cluster these functions into MetaCyc metabolic pathways (Caspi et al. 2018). In contrast to the taxonomic results, we observed no clear functional differences between host species (supplementary fig. S9 and table S2, Supplementary Material online). The most abundant pathways were shared across individuals and host species and involved essential metabolic processes, such as energy production and biomolecule synthesis (supplementary fig. S10, Supplementary Material online). Pathways contributing to sample separation in a principal component analysis (PCA) included those involved in core biosynthesis and metabolism, biosynthesis of bacterial cell wall components, and degradation of plant-associated metabolites (supplementary fig. S9, Supplementary Material online). The caries bear Ua9 contained a distinct functional profile (supplementary fig. S9 and S10, Supplementary Material online) with metabolic pathways relating to carbohydrate fermentation and acid production (fig. 2*c*). Enzymes encoded by *L. casei* group bacteria and mutans streptococci substantially contributed to one of these pathways (P461-PWY).

### Antimicrobial Resistance Genes Are Present in Wild Animal Microbiomes

**Fig. 3.**
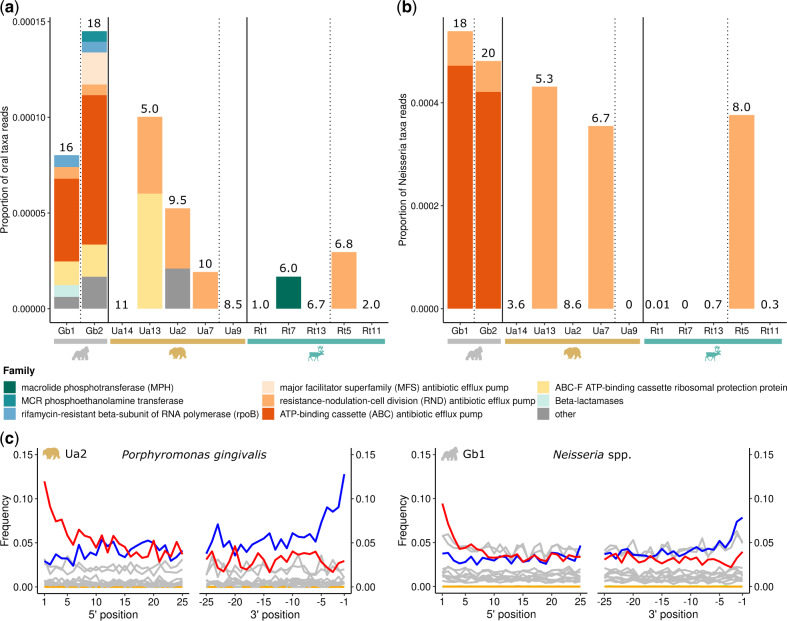
AMR genes can be recovered from historical dental calculus of wild animals. (*a*) Proportion of AMR genes identified in contamination-filtered reads mapping to oral bacteria (listed in supplementary table S4, Supplementary Material online) in each sample. (*b*) Proportion of chromosomally encoded AMR genes identified in contamination-filtered reads mapping to *Neisseria* species in each sample. Samples are grouped by host species and ordered by year with pre-1940 samples (preantibiotics era) separated from post-1940 samples by dashed vertical lines. The eight most abundant AMR gene families are shown, with the remainder grouped into “other.” Number of reads (in ten thousands) mapping to oral bacteria is shown above the bars for each sample in (*a*), and number of reads (in thousands) mapping to *Neisseria* taxa is shown in (*b*). (*c*) Postmortem DNA damage patterns of reads mapping to example oral bacteria: *Porphyromonas gingivalis* from bear Ua2 and all reads mapping to a set of *Neisseria* spp. reference genomes from gorilla Gb1. Frequency of C-to-T substitutions in reads compared with the reference is shown in red and G-to-A substitutions in blue. All other substitutions are shown in gray, insertions in purple, deletions in green, and soft clipping in orange, representing background noise. Misincorporation plots were generated by mapDamage.

The presence of bacteria carrying antimicrobial resistance (AMR) genes has been documented in the human oral microbiome (Xie et al. 2010; Warinner et al. 2014). We therefore investigated whether oral microbial communities of wild animals contain AMR genes and whether their abundance differs across host species. To this end, we investigated the diversity and abundance of AMR genes in oral bacteria (supplementary table S4, Supplementary Material online) detected in our samples, by blasting contamination-filtered reads mapping to oral bacteria (assigned by MALT) against the Comprehensive Antibiotic Resistance Database (CARD) (Jia et al. 2017). The top match for each read was assigned to its respective gene family under the Antibiotic Resistance Ontology (ARO). We repeated the process targeting AMR genes chromosomally encoded by *Neisseria*, a host-associated genus of bacteria with commensal species in the oral microbiome, including dental plaque, of humans and pets (Dewhirst et al. 2012; Heydecke et al. 2013; Dewhirst et al. 2015; Lloyd-Price et al. 2017). In both oral bacteria and the targeted *Neisseria* analysis, reads mapping to AMR genes were detected in all three host species (fig. 3*a* and *b*), though they were more abundant in samples from bears and gorilla. Genes with similarity to antibiotic efflux pumps were the most commonly observed gene families. Oral taxa, including those related to *Neisseria*, exhibited characteristic deamination patterns consistent with postmortem DNA damage in authentic historical taxa (fig. 3*c*).

### De Novo Metagenome-Assembled Genome Recovery

As a complement to reference-based microbial taxonomic assignment, we used de novo metagenome-assembled genome (MAG) assembly techniques to recover 22 medium-quality draft MAGs from one gorilla, three bear, and three reindeer specimens (supplementary table S5, Supplementary Material online). Eight draft MAGs from the bears and reindeer were estimated to be >90% and showed typical DNA damage patterns (supplementary fig. S11, Supplementary Material online; for a technical discussion of some aspects of the damage patterns, see supplementary table S6, Supplementary Material online). Six of these draft MAGs were classified as taxa related to oral bacteria (*Lactobacillus*, *Streptococcus*, and *Haemophilus*) (supplementary table S5, Supplementary Material online), including the cariogenic *L. casei* group and the mutans streptococci group from Ua9. The other two draft MAGs were recovered from one bear specimen but lacked similarity to characterized reference genomes. They thus possibly represent novel bacteria specific to the bear oral cavity. Although MAGs have been recovered from historical samples using reference-based methods (Zhou et al. 2018; Achtman and Zhou 2019), our study is one of the first to report successful de novo recovery of draft MAGs from historical samples. We therefore explored at what sequencing depth such de novo analyses become feasible (supplementary fig. S12, Supplementary Material online). As expected, shallower sequencing depth was required to reach “higher quality” (>90% genome completeness and <5% strain contamination [Bowers et al. 2017; Parks et al. 2017]) for highly abundant taxa, such as the MAGs related to *L. casei* and mutans streptococci recovered from Ua9 (fig. 2*b*).

### Dental Calculus as a Source of Dietary Information

**Fig. 4.**
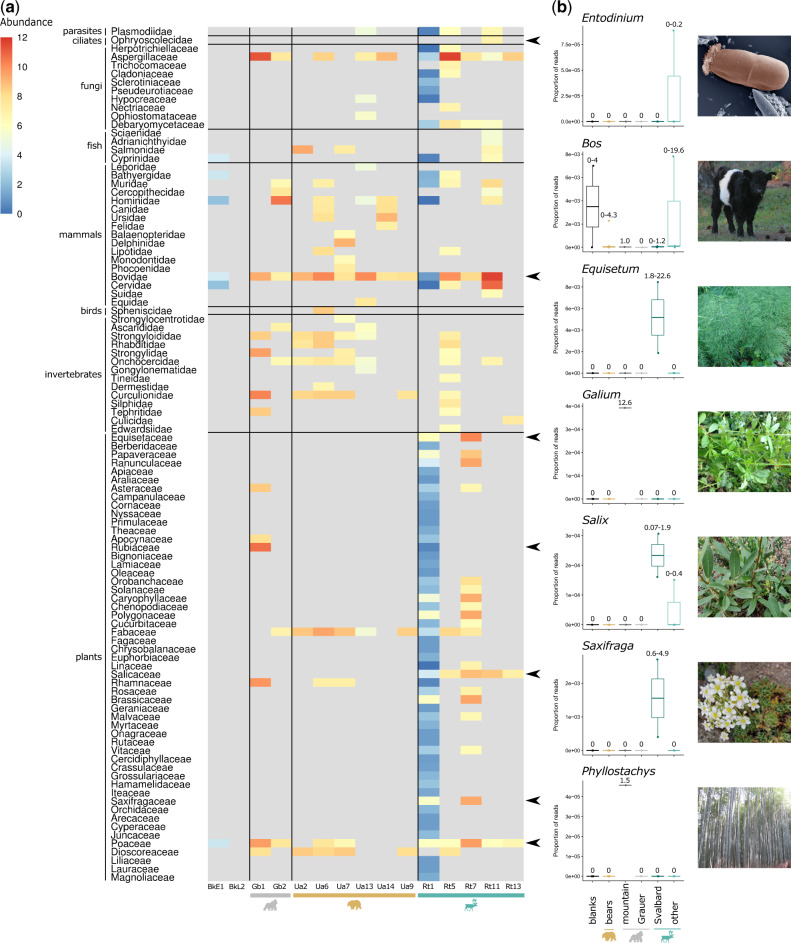
Host diet can be inferred from dental calculus. (*a*) MALT/MEGAN CLR normalized abundance of eukaryotic reads at the family taxonomic level. Taxa that were not detected in a sample are colored gray. Broad groups of eukaryotes are designated by horizontal black lines. Black arrows indicate selected families for which genus-specific relative abundances are plotted in (*b*). (*b*) Proportion of reads mapping to specific eukaryotic genera in different host species, including blank controls (visualized as Tukey boxplots). Gorilla samples are divided into the two subspecies (mountain and Grauer’s gorillas) and reindeer are divided into the high Arctic Svalbard ecotype and “other” ecotypes (mountain and forest) to illustrate population-specific differences in dietary components. The minimum and maximum number of reads mapping (in thousands) to each genus for the samples in each host category is shown above the boxplots. The *Bovidae* genus *Bos* is included as an example of spurious mappings, due to the presence of reads in samples from multiple host species and blank controls. Genera boxplots are ordered by family as indicated by the black arrows in (*a*). Image credit: *Entodinium caudatum* photo by Sharon Franklin and colorization by Stephen Ausmus, USDA Agricultural Research Service (www.ars.usda.gov/oc/images/photos/feb06/d383-2/, last accessed June 19, 2019) and other images by Katerina Guschanski and Jaelle Brealey.

To explore the potential of dental calculus to provide insights into the dietary composition of each host species, we taxonomically profiled all eukaryotic reads (excluding those mapping to the host and human during data preprocessing) from our samples using MALT (Vågene et al. 2018) and MEGAN (Huson et al. 2016) against the NCBI nucleotide database (fig. 4*a*). We observed a number of likely spurious mappings and/or contamination, such as to the bovine genus *Bos*, which showed mapping of reads from all three host species and the negative controls (fig. 4*b*). It is possible that this contamination originated from the presence of bovine serum albumin in one of the buffers used during library preparation (see Materials and Methods). In bears, only few plant-based dietary components were identified and we found no clear patterns for mammalian or invertebrate putative dietary items, as similar taxa were also present in some reindeer and/or gorilla samples, which are not expected to consume animals. We were able to infer population-specific dietary characteristics in gorilla and reindeer samples, although in many cases the taxa identified in our analyses are likely close relatives to the consumed species, which are not well represented in the reference databases. For example, among reads that mapped to the *Poaceae* family in the mountain gorilla, ∼28% mapped to *Phyllostachys*, a genus of Asian giant timber bamboo (fig. 4*b* and supplemental table S10, Supplementary Material online). Mountain gorillas are known to consume a related *Arundinaria alpina* bamboo (Grueter et al. 2013), the genome of which is not currently available. We also identified *Galium* vines in the mountain gorilla (fig. 4*b*), consistent with their known dietary preferences (Grueter et al. 2013; Galbany et al. 2016). *Salicaceae* plants, for example, *Salix* (willows) were identified in all reindeer (fig. 4*b*), consistent with the known browsing behavior of these animals (Skogland 1980). A number of Arctic plants were identified in the Svalbard reindeer (Rt1 and Rt7) that are known or likely components of the high Arctic reindeer diet, including *Saxifraga* spp. and *Oxyria* spp. (Staaland et al. 1993) (fig. 4*b*). Furthermore, reads assigned to rumen ciliates from the Ophryoscolecidae family (*Entodinium caudatum* and *Epidinium ecaudatum*) were identified in one of the reindeer samples (Rt11, fig. 4*b*).

### Recovery of Host Genomic Profiles

**Fig. 5.**
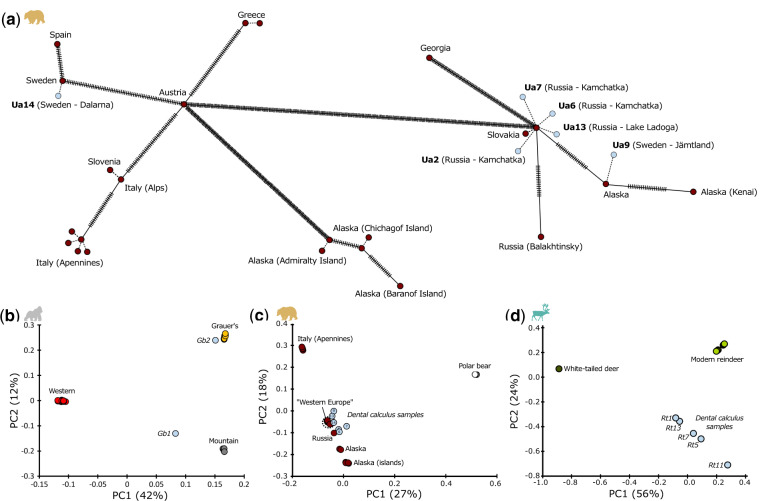
Host population genetic structure can be reconstructed from dental calculus. (*a*) mtDNA haplotype network for brown bears. Each circle represents a sample, with ticks on the connecting lines showing the number of base pair substitutions between the haplotypes. Dotted lines represent identical haplotypes or in the case of dental calculus samples (shown in light blue), the predicted most closely related haplotype. Sample labels include country and locality of specimen collection. Note that dental calculus samples of Swedish bears cluster with both eastern (Russia) and western (Spain) modern genomes, consistent with the history of postglacial colonization of Sweden. (*b*–*d*) PCA of modern high-coverage genomes and low-coverage dental calculus samples projected onto the modern genomes for gorilla (*b*), brown bear (*c*), and reindeer (*d*). Samples cluster together with their respective species. Dental calculus gorilla samples cluster most closely to their subspecies of origin. Dental calculus brown bear samples from western Europe and Russia cluster with the modern genomes from the “western Europe” clade (consisting of Spain, Greece, Slovenia, Italy [Alps], Sweden, and Slovakia) and the Russian clade, respectively. Dental calculus reindeer samples are clearly separated from the white-tailed deer outgroup.

In addition to microbial remains, dental calculus has also been successfully used as a source of host DNA (Ozga et al. 2016; Mann et al. 2018). We therefore identified host DNA preserved in dental calculus samples (including bear Ua6 excluded from the microbial analyses) by mapping the reads to reference genomes of the host species’ closest phylogenetic relatives. For mitochondria, 2.6–99.7% (median 91.2%) of the genome was covered by at least one read in each study sample with the coverage depth ranging from 0.02 to 174× (supplementary table S7, Supplementary Material online). Indeed, the high abundance of host reads in some samples allowed us to reconstruct complete mitochondrial genomes from five specimens (supplementary table S7, Supplementary Material online). For nuclear genomes, 0.004–21.3% (median 0.346%) were covered by at least one read, with maximum genome-wide coverage of 0.3×. We compared the recovered genomic profiles with published genomes from the same species. Mitochondrial haplotypes could be placed within species-specific mitochondrial networks (fig. 5*a* and supplementary figs. S13 and S14, Supplementary Material online). Projection of low-coverage host nuclear genomes onto the PCA space precalculated from high-quality published genomes clearly assigned all study samples to their correct species (reindeer), subspecies (gorilla), and even to broader geographic populations of origin (brown bear) (fig. 5*b*–*d*).

## Discussion

### Dental Calculus Contains Host Species-Specific Oral Microbiome Signatures

We successfully recovered an oral microbiome signature from museum-preserved dental calculus samples of all three studied host species (fig. 1). The recovered microbial communities differed by host species, likely reflecting the evolutionary distance and ecological differences between them, similar to how host evolutionary relationships and diet have been shown to structure other host-associated microbiomes (for example, Ley et al. 2008; Groussin et al. 2017). Generally, the proportion of identifiable oral microbiome signature was highest in the two gorilla individuals, likely the result of improved taxonomic classification due to the close phylogenetic relationship between gorillas and the source of the oral microbiome database (humans). Taxa related to *Streptococcus*, a genus known to colonize the mammalian oral cavity (Dewhirst et al. 2012, 2015; Lloyd-Price et al. 2017) were frequently observed in both gorilla and healthy bear samples. In contrast, reindeer tended to have lower proportions of oral bacteria compared with gorillas and the greatest proportion of uncharacterized microbial taxa (supplementary fig. S6, Supplementary Material online). Reindeer also had the lowest alpha diversity of the three host species (supplementary fig. S8, Supplementary Material online) and the lowest detected prevalence of AMR (fig. 3), which may at least partly reflect poor representation of reindeer oral taxa in the reference database. Instead, a number of reindeer samples contained taxa related to rumen-associated microbes, such as *Methanobrevibacter* species of bovine and ovine rumens (Janssen and Kirs 2008) and *Ophryoscolecidae*, a family of rumen ciliates that are important facilitators of digestive processes in ruminants (Zeitz et al. 2012). Ruminants regurgitate large amounts of rumen material into the oral cavity when chewing cud, and a study in sheep has found that oral swabs contain a proportion of rumen-associated microbes (Kittelmann et al. 2015). Thus, it is possible that the dental calculus of ruminants captures a subset of the rumen microbiome, which may also contribute to the comparatively lower proportion of oral taxa we observed in reindeer dental calculus.

Despite the clear taxonomic distinctions, we found no functional differences in the oral microbiome of the studied host species (supplementary fig. S9, Supplementary Material online). Functional conservatism despite differences in community composition has been observed in diverse ecosystems, including the human microbiome (Huttenhower et al. 2012; Lloyd-Price et al. 2017). However, these results may be driven by limitations of the functional reference databases, which are mostly centered on global mechanisms (Prakash and Taylor 2012) and do not allow for more fine-grained functional characterization. The use of translated nucleotide searches against a protein database, one of the key steps in the HUMAnN2 functional classification pipeline used here, may also not be optimal for ancient metagenomes (Eisenhofer and Weyrich 2019), potentially leading to an underestimation of the functional content of our samples.

### Oral Pathogens in Wild Animals

Human dental plaque contains a number of microorganisms that under certain conditions can contribute to oral diseases, such as dental caries or periodontitis (Takahashi and Nyvad 2011; Costalonga and Herzberg 2014). For example, increases in dietary sugars may promote growth of acid-producing bacteria like *Streptococcus mutans* in plaque, leading to demineralization of tooth surfaces and the initiation or progression of dental caries (Takahashi and Nyvad 2011). Oral pathologies have been observed in domestic, captive, and wild animals (Gorrel 1998; Kennedy et al. 2016), including wild North American black bears (Manville 1990) and captive brown bears (Wenker et al. 1998). We observed evidence of dental caries in two of six studied bear specimens. In the sample taken from a caries lesion (Ua9), we identified high abundance of taxa related to known cariogenic bacteria in humans, that is, *L. casei* and mutans streptococci (Tanzer et al. 2001; Neves et al. 2017), through both reference-based taxonomic assignment with Kraken2 (fig. 2*b*) and de novo MAG assembly (supplementary table S5, Supplementary Material online). From this sample, we also identified metabolic pathways that were generally involved in carbohydrate fermentation and acid production, functions commonly performed by bacteria that colonize the oral cavity and are also associated with the emergence and progression of dental caries (Takahashi and Nyvad 2011). Correspondingly, enzymes encoded by *L. casei* group bacteria and mutans streptococci substantially contributed to one of these pathways in Ua9. In contrast, samples taken from healthy teeth (irrespective of caries presence in the specimen) had lower abundances of these potentially cariogenic bacteria and associated pathways. The biogeography of the oral microbiome in humans has been found to be site specific (Mark Welch et al. 2016; Proctor et al. 2018), and within a diseased individual, the oral microbiome has been shown to differ between healthy and carious sites (Richards et al. 2017). The periodontal “red complex” pathogens (*P. gingivalis*, *Treponema denticola*, and *Tannerella forsythia*) (Socransky et al. 1998) were identified in most bear and gorilla samples without signs of oral disease, consistent with findings in humans, where these bacteria are normal members of the mature plaque biofilm (Kolenbrander et al. 2002; Velsko et al. 2019). However, our current inferences about the oral taxa associated with bear dental caries are limited by our small sample size (*n* = 1), the reliance on human-biased microbial reference databases and the fact that detection of potential pathogens, particularly via DNA techniques, does not imply causation.

### AMR in the Wild Animal Oral Microbiome

Since human mass production of antibiotics started in the 1940s, AMR has been increasing and now poses a serious global public health threat (Crofts et al. 2017; Thorpe et al. 2018). It is therefore imperative to identify sources and transmission routes of AMR. Animal populations can act as reservoirs and contribute to the spread of AMR elements to humans, yet we know very little about AMR dynamics in wildlife (Allen et al. 2010; Arnold et al. 2016; Vittecoq et al. 2016). Furthermore, AMR is a normal function of natural environments, including host-associated microbiomes, which makes it challenging to distinguish between anthropogenic and natural sources (Allen et al. 2010; Nesme et al. 2014). Historical samples spanning from before the industrial-scale production of antibiotics in the 1940s to today are thus needed to fully elucidate the role of wildlife in AMR dynamics. Although we cannot make any inferences about possible changes in AMR potential through time with our limited sample numbers, we detected AMR genes in dental calculus microbial communities both before and after the 1940s (fig. 3). Our analyses are thus in line with previous studies on historical human-associated and permafrost microbiomes that demonstrate that many of the underlying molecular mechanisms conferring resistance to (modern) antibiotics have existed in the environment long before mass antibiotics production (D’Costa et al. 2011; Warinner et al. 2014). For example, the most common AMR gene families identified in our analyses were related to antibiotic efflux pumps. Many efflux pumps, for example, the resistance-nodulation-division family, have generalized roles pumping out toxins such as heavy metals, and thus their function as agents conferring AMR may be secondary to their primary role of providing tolerance to toxic compounds (Poole 2005). Our investigation opens doors for the use of dental calculus as a tool to study “natural” AMR and its evolution through time in wild animals from diverse geographic locations, and for determining the potential of wildlife to serve as reservoirs for clinically relevant AMR factors.

### Beyond Microbes: Dietary and Host DNA Recovery from Metagenomics Data

Dental calculus has been used as a source of both dietary information and host genetic material previously in primates (Warinner et al. 2014; Mann et al. 2018; Modi et al. 2020). Dietary reconstruction from shotgun sequencing reads alone is challenging and must be interpreted with caution (Dickson et al. 2017; Charlier et al. 2019). However, we were able to identify population-specific dietary characteristics in gorilla and reindeer samples (fig. 4). Recovery of putative dietary components was particularly successful from reindeer calculus, where we also identified microbial functional pathways that may be related to dietary degradation of plant matter (supplementary fig. S9, Supplementary Material online). However, identification of dietary components at the species or even genus level was low, since often only a small number of reads mapped to the reference genome and many plant taxa are not well represented in the reference genome databases. Combining dental calculus DNA analyses with microfossil investigations, ancient proteomics, or DNA extractions specifically designed for plant microremains will likely improve future dietary characterization (Mowat and Heard 2006; Warinner et al. 2014; Power et al. 2015; Cristiani et al. 2018; Modi et al. 2020).

Both host whole nuclear genome and whole mitochondrial genome reconstruction from dental calculus samples have generally required target enrichment techniques (Ozga et al. 2016; Ziesemer et al. 2019; Modi et al. 2020). We were able to reconstruct complete mitochondrial genomes from five of our 18 specimens and recover low-coverage nuclear genomes directly from the shotgun metagenomics data (fig. 5). In the bears, the mitochondrial haplotype network reflected known differences in the colonization history of Scandinavia from western Europe and Russia (Xenikoudakis et al. 2015). In all three host species, the low-coverage nuclear genomes were of sufficient quality to allow identification of species, subspecies, and even broad geographic origin. Differences in study methodology and dental calculus morphology (fig. 1*a*) could explain why our samples generally contained more host DNA than dental calculus derived from humans (Mann et al. 2018).

### Dealing with Current Limitations: Contamination and Database Biases

Dental calculus has been little explored outside primates, yet it can be a treasure trove of information about evolutionary and ecological processes of the host and its oral microbiome for many different species. However, research on microbiomes from the past, including from dental calculus, is hindered by a number of challenges. We have established rigorous laboratory and computational procedures for overcoming the problem of contamination, which affects all historical genomic and metagenomic studies (Key et al. 2017). However, despite these measures, SourceTracker results indicated that some proportion of human skin taxa remained in our samples (fig. 1*b*). Several bacterial species colonize multiple niches within the host (Huttenhower et al. 2012; Lloyd-Price et al. 2017), which can obscure distinction of a genuine signal from a likely contaminant. For example, *Streptococcus mitis*, *Staphylococcus epidermidis*, and *Corynebacterium matruchotii* are found in the human mouth, nostrils, and skin (Huttenhower et al. 2012). This limitation is not specific to our data set but poses greater problems for studies based on historical samples that are expected to be subject to contamination.

The other common limitation faced by our and many other studies is the reliance on microbial reference databases. These databases are heavily biased toward microbial species with medical or agricultural significance (Rinke et al. 2013), restricting read-based analyses of metagenomics data from nonhuman hosts. On average, 72% of the nonhost reads in our samples could not be assigned taxonomy by Kraken2 (range 31–90%, supplementary table S9, Supplementary Material online). Of the microbial taxa that could be identified, a large proportion remained unassigned to any source microbiome by SourceTracker (supplementary fig. S6, Supplementary Material online). Although some of these taxa may be members of other microbial communities not included as a source in our analysis, we expect that by studying a novel environment (the nonhuman oral microbiome), we will encounter unique microbial taxa. In the absence of a dedicated reference database from the study species, a complementary approach is de novo MAG assembly (Rinke et al. 2013; Parks et al. 2017). Given the fragmented and damaged nature of ancient DNA, this technique poses great challenges for historical microbiome studies. However, our study demonstrates that with deeper sequencing MAG recovery may be able to complement read-based analyses of historical microbiome samples. Because the likelihood of recovering high-quality draft MAGs strongly depends on the sequencing depth and complexity of the metacommunity (a factor that cannot be established a priori), we suggest that MAG assembly could be attempted concurrently to reference-based mapping, particularly when reference-based mapped results suggest that a sample is dominated by a taxon of interest. Such approaches are particularly important in nonmodel species, where reference database bias is a problem.

### The New Addition to the Evolutionary Toolkit

With the development of high-throughput sequencing techniques and methodological advances in metagenomic analyses of ancient samples, the time is ripe to investigate environmental and host-associated microbial communities from the past. The temporal perspective provided by historical and ancient samples allows us to study many fundamental evolutionary processes, including those with direct relevance to human and ecosystem health. Our work describes a rigorous roadmap for the analysis of historical microbiomes and illuminates a multitude of biological questions that can benefit from the study of dental calculus remains. We demonstrate that a single sample source can be used to link the host microbial community to host genetics, diet, and even disease, although larger sample sizes are needed to substantiate the biological inferences of our preliminary findings. Although the versatility of dental calculus has been already detailed in humans (Warinner et al. 2014), our study establishes dental calculus as a tool for evolutionary exploration from a comparative perspective. Questions of interest include the evolution of the host-associated microbiome through periods of external environmental change, the invasion of new habitats, changes in competitive regimes, or alterations in host population demography and genetic diversity. Temporal sampling of dental calculus from past populations also provides insights into oral disease emergence and the progression of AMR in host-associated microbiomes. These processes can be of interest to both evolutionary biologists and the public health sector, since wild animal populations can act as sources and reservoirs for emerging zoonotic pathogens (Reperant et al. 2016; Gong and Bao 2018) and contribute to the spread of AMR (Arnold et al. 2016; Vittecoq et al. 2016). In addition, our dietary results indicate that dental calculus can be used to infer population-specific dietary characteristics, particularly if complemented with microfossil analysis from the same material and stable isotope analysis of teeth or bones (Mowat and Heard 2006; Power et al. 2015; Cristiani et al. 2018), which can be extended to extinct species (e.g., Henry et al. 2011). As we have demonstrated, metagenomic analyses of dental calculus can be performed on a diverse range of mammalian species, which will allow investigation into many different questions in ecology and evolution.

## Materials and Methods

### Sample Collection

Dental calculus was collected from two eastern gorilla (*Gorilla beringei*) specimens from the Royal Museum for Central Africa (Brussels, Belgium), as well as five reindeer (*R. tarandus*) and six brown bear (*U. arctos*) specimens from the Swedish Natural History Museum (Stockholm, Sweden). Skulls were macroscopically examined for dental calculus deposits and evidence of oral diseases. Calculus was removed from the surfaces of the teeth with disposable sterile scalpel blades and deposited in sterile microcentrifuge tubes. In individuals without macroscopic signs of oral disease, calculus deposits from multiple teeth were pooled, whereas in individuals with dental caries we sampled from caries lesions separately. Dental caries were only observed in the bear specimens.

### Sample Processing and DNA Extraction

All laboratory protocols were performed in a dedicated ancient DNA laboratory following stringent procedures to minimize contamination (Key et al. 2017). Initially, calculus samples were processed without surface decontamination. We then tested the effect of surface decontamination on the two gorilla samples (Supplementary Material online). Based on real-time polymerase chain reaction (PCR) of libraries prepared from these samples (see below), we continued with a surface decontamination procedure consisting of UV light exposure (10 min at 245 nm) followed by a wash in 500 µl of 0.5 M EDTA for 30 s (Ozga et al. 2016) for all subsequent calculus samples, and the pellet was taken forward for DNA extraction. Samples from both the initial processing without surface decontamination and the later processing with the UV + EDTA wash treatment were included in the final analysis (supplementary table S1, Supplementary Material online). DNA extractions were performed using <5–20 mg of dental calculus per sample, following a silica-based method (Dabney et al. 2013). Briefly, samples were incubated overnight at 37 °C in extraction buffer (0.45 M EDTA, 0.25 mg/ml Proteinase K). The DNA from the supernatant was combined with binding buffer (3 M sodium acetate, 5 M guanidine-hydrochloride, 40% [v/v] isopropanol, and 0.05% [v/v] Tween-20) and processed through the spin columns from High Pure Viral Nucleic Acid Large Volume kits (Roche, Switzerland). Purified DNA was eluted in either 45 µl of EB buffer (10 mM tris-hydrochloride [pH 8.0]) (Qiagen, the Netherlands) or 45 µl of TE buffer (10 mM tris-hydrochloride [pH 8.0], 1 mM EDTA), both supplemented with 0.05% (v/v) Tween-20 (supplementary table S1, Supplementary Material online). Samples were processed in five extraction batches that each contained additional historical calculus samples not included in this study (host species were randomly distributed across batches), with two extraction blanks included per batch. All ten blanks were carried through library preparation.

### Library Preparation and Sequencing

Double-stranded Illumina libraries were prepared following Meyer and Kircher (2010), and we included a double-barcoding double-indexing strategy to guard against index hopping and retain absolute certainty about sample of origin (Rohland et al. 2015; van der Valk, Vezzi, et al. 2019). Briefly, blunt-end repair reactions were performed using 20 µl of each extract (note this step was performed with Tango Buffer) (ThermoFisher Scientific, USA), which contains 0.1 mg/ml bovine serum albumin) and purified using MinElute columns with elutions in 22 µl of EB buffer (Qiagen, the Netherlands). Adapters containing inline 7-bp barcodes (supplementary tables S1 and S8, Supplementary Material online) were ligated to both ends of the blunt-ended DNA, which was subsequently purified with MinElute columns and eluted in 22 µl of EB buffer. After the adapter fill-in reaction, *Bst* 2.0 polymerase (New England BioLabs, USA) was inactivated with a 15-min incubation at 80 °C. Libraries were prepared in four batches, again randomizing across host species and including the ten extraction blanks, with 1–2 library blanks per batch. The adapter-ligated libraries were quantified using a real-time PCR assay with preHyb primers (Rohland et al. 2015) (supplementary table S8, Supplementary Material online) and the estimated fragment number was used to approximate the number of indexing PCR cycles needed for sequencing. All extraction and library blanks were consistently lower in DNA content than samples, as measured by real-time PCR, thus one extraction blank and one library blank were randomly selected for subsequent indexing and sequencing. Libraries were double-indexed with unique P5 and P7 indices so that each sample had a unique barcode-index combination (supplementary table S1, Supplementary Material online). Indexing PCR reactions were performed with 18 µl of adapter-ligated library in 50 µl reactions, with 1 µl PfuTurbo C_x_ hotstart polymerase (2.5 U/µl, Agilent Technologies, USA), 5 µl 10× PfuTurbo C_x_ reaction buffer, 0.5 µl dNTP mix (25 mM) and 1 µl of each indexing primer (10 µM). After an initial incubation for 2 min at 95 °C, 12 cycles of 30 s at 95 °C, 30 s at 59 °C, and 1 min at 72 °C were performed, followed by a final step of 10 min at 72 °C. Reactions were purified with MinElute columns and eluted in 10 µl of EB buffer supplemented with 0.05% (v/v) Tween-20. The indexed libraries were quantified using a real-time PCR assay with i7 and i5 indexing primers (Rohland et al. 2015) (supplementary table S8, Supplementary Material online) and library DNA fragment length distribution was determined by the 2200 TapeStation system. The mean fragment length after library preparation and excluding the 148-bp adapter sequences was 70 bp, similar to what has been observed in previous historical sequencing libraries (van der Valk et al. 2017; Mann et al. 2018). Five microliters of each sample library were pooled along with a randomly selected extraction blank and library blank. Size selection was performed on the pooled library with AMPure XP beads (Beckman Coulter, USA), selecting for fragments ∼100–500 bp in length, and the purified library eluted in 36 µl of EB buffer. The final pooled library was quantified using both a Qubit High Sensitivity fluorometer and the 2200 TapeStation system. The pooled library was first sequenced by SciLifeLab Uppsala on two lanes of the Illumina HiSeq 2500 using paired-end 125-bp read length v4 chemistry, followed by an additional two lanes on the Illumina HiSeq 2500 in rapid mode using paired-end 100-bp read length v2 chemistry.

### Data Processing

The data processing and analysis steps are summarized in supplementary figure S1, Supplementary Material online. Sequencing data were demultiplexed and assigned to each sample with an in-house python script based on the unique combination of barcodes and indices. Overlapping paired-end reads were merged and adapters and low quality terminal bases (phred scores ≤30) were removed with AdapterRemoval v2.2.2 (Schubert et al. 2016). Barcode sequences were removed from the 5′ and 3′ ends of merged reads with an in-house python script. Forward reads from the unmerged read pairs (i.e., pairs that did not contain overlapping regions of at least 11 bp between the forward and reverse reads) were also retained for analyses. The 5′ barcode was removed with an in-house python script and the 3′ barcode with any remaining adapter sequence removed with AdapterRemoval. Reads from the two lanes within the same sequencing run were concatenated into a single file per sample. Merged reads from the two separate runs were also concatenated into a single file per sample. Reads with a length <30 bp were filtered out with AdapterRemoval and reads with mean base quality <30 were filtered out with PrinSeq-Lite v0.20.4 (Schmieder and Edwards 2011). Duplicate reads were removed by randomly keeping one read among those reads having an identical sequence. The Illumina sequencing control phage PhiX was spiked into our sequencing runs and has been reported to have been erroneously integrated into many microbial reference genomes (Mukherjee et al. 2015; Hooper et al. 2019). Reads were therefore mapped to PhiX (accession: GCA_000819615.1) with bwa mem v0.7.17 (Li and Durbin 2009; Li 2013) and the unmapped reads retained with SAMTools v1.9 (Li et al. 2009) and BEDTools v2.21.0 (Quinlan and Hall 2010). To remove reads originating from the host organism and from human contamination, we mapped all reads in a sample to a combined reference consisting of the human genome (Schneider et al. 2017) (RefSeq accession: GCF_000001405.38) and the respective host genome (GCF_000151905.2 [*Gorilla gorilla gorilla*] [Scally et al. 2012], GCF_003584765.1 [*U. arctos horribilis*] [Taylor et al. 2018], and GCA_004026565.1 [*R. tarandus*]) with bwa mem. The unmapped reads were retained with SAMTools for downstream microbial taxonomic analyses.

### Taxonomic Assignment

Merged and unmerged unmapped reads were assigned taxonomy using the *k*-mer based classifier Kraken2 v2.0.7 (Wood and Salzberg 2014) with the standard Kraken2 database (all archaea, bacteria, viruses, and the human genome in RefSeq; built March 1, 2019) and default parameters. The algorithm underlying Kraken’s classification has been shown to perform well in ancient metagenomes (Velsko et al. 2018). We used Kraken-biom (github.com/smdabdoub/kraken-biom, last accessed November 19, 2018) to extract the summarized number of reads assigned at the genus and species levels. These assignments were taken into R (R Core Team 2020) for further processing (see below; final data set provided in supplementary table S9, Supplementary Material online). Across all dental calculus samples, on average 84% of reads could be assigned species-level taxonomy (range 74–95%, supplementary table S9, Supplementary Material online). We also taxonomically binned the contamination-filtered reads (see below) against a wider database by alignment with MALT v0.4.0 (Vågene et al. 2018) (parameters as per Mann et al. [2018]: semiglobal alignment, 85% minimum identity threshold, 0.01% minimum support threshold, and a top percent value of 1.0) against an index built from the entire NCBI nucleotide database (ftp.ncbi.nlm.nih.gov/blast/db/FASTA/nt.gz, modified May 31, 2018) using a step size of 4 to reduce index size. The results were viewed in MEGAN Community Edition v6.10.5 (Huson et al. 2016) (supplementary table S10, Supplementary Material online) and were used in specific analyses as detailed below and summarized in supplementary figure S1, Supplementary Material online.

### Identifying Contamination

We used several complementary approaches to identify and remove contaminating bacterial taxa from the Kraken taxonomy assignments.

#### SourceTracker Analysis

Potential contribution of source microbiomes to samples was estimated with SourceTracker v1.0 (Knights et al. 2011) in R, using the Kraken2 genus- and species-level assignments. Source sequencing reads were processed through the same pipeline as sample reads, and included soil (Johnston et al. 2016), human skin (Oh et al. 2014), human gut (Huttenhower et al. 2012; Lloyd-Price et al. 2017), human supragingival plaque (Huttenhower et al. 2012; Lloyd-Price et al. 2017), human medieval dental calculus (Mann et al. 2018), and laboratory reagent (Salter et al. 2014) microbiomes (supplementary table S11, Supplementary Material online).

#### Fragment Length

Given our library fragment length distribution peak at 70 bp, consistent with historical degraded DNA, and our paired-end sequencing approach of 100 or 125 bp, we expected that the majority of reads stemming from authentic historical microorganisms would be successfully merged by AdapterRemoval (supplementary fig. S5, Supplementary Material online). In contrast, modern contaminating taxa with longer fragment lengths should be predominantly found among the unmerged reads. We therefore compared the overall taxonomic composition as well as the raw read counts of each taxon between the merged and unmerged reads on a per sample basis (supplementary fig. S5, Supplementary Material online). Taxa that were overrepresented in the unmerged reads in at least one sample were filtered out as putative contaminants. It should be noted that the unmerged reads were only used for this contaminant identification step and did not contribute to our inferences about microbial community composition, function, MAG reconstruction, etc.

#### Statistical Identification of Contaminants

Contamination derived from environmental sources (including the laboratory) is expected to be present in samples at approximately similar absolute amounts and will therefore be disproportionally more abundant in samples with low numbers of total sequenced reads (Lusk 2014; Salter et al. 2014). This concept has been implemented in the R package decontam (Davis et al. 2018), which was used to identify and remove all taxa that showed an inverse relationship between taxon abundance and total number of sequences included in the sequencing pool per sample, as estimated by real-time PCR.

#### Presence in Blanks

SourceTracker analysis demonstrated that the two blank samples contained taxa associated with soil and human skin microbiomes (supplementary fig. S3, Supplementary Material online). However, low levels of sample cross-contamination are common during laboratory processing. Conservatively filtering out all taxa observed in the blanks might remove genuine signals. We therefore screened all taxa that were present in the blanks against the Human Oral Microbiome Database (HOMD) (www.homd.org/index.php? name=Download&file= download&table=tt&format=html, accessed January 22, 2019) (Chen et al. 2010). Taxa that were not present in HOMD were classified as contaminants and removed from further analysis.

#### Removal of Contaminants from Reads

For all downstream analyses, merged reads mapping to all species-level taxa (bacteria, archaea, and viruses) identified as putative contaminants were removed from the fastq files. To this end, one reference genome for each taxon was downloaded from GenBank (supplementary table S12, Supplementary Material online; accessed April 11, 2019) and merged reads were mapped to the reference genomes with bwa mem v0.7.17, relaxing the mismatch parameter (-B) to 3 in order to map reads from closely related strains. Only unmapped, merged reads were passed onto further analyses.

### Microbial Analyses

#### Genome Size Normalization

Taxa with larger genomes will generally contribute more sequencing reads to a library, biasing read-based abundance estimates (Frank and Sørensen 2011). To account for this bias, the number of reads per taxon within a sample was normalized by dividing by the estimated average genome size of each respective taxon. Estimated average prokaryotic and viral genome sizes were calculated using publicly available genome sizes from the RefSeq database (accessed February 15, 2019) (O’Leary et al. 2016). In cases where no genome size data were available for a given species, the average genome size of taxa in that genus was used.

#### Abundance Filtering

Taxa present at <0.03% relative abundance (normalized read count divided by sum of normalized read counts in a sample) were removed, as filtering of low-abundance species reduces false-positive taxonomic assignments (Velsko et al. 2018). The filtering threshold was selected by testing a series of thresholds commonly applied in metagenomics studies, ranging from 0.01% to 0.1% (Peabody et al. 2015) (supplementary fig. S15, Supplementary Material online). From this analysis, we identified the threshold (0.03%) that yielded a microbial community with a complexity which was most similar to what has been observed in other dental calculus (Mann et al. 2018) and oral microbiome studies (Dewhirst et al. 2010, 2012, 2015; Kilian et al. 2016) (i.e., ∼100–300 taxa). This table of genome size normalized, contaminant-filtered, and abundance-filtered taxa counts (supplementary table S9, Supplementary Material online) was used for subsequent microbial analyses.

#### Abundance Normalization

In high-throughput microbiome sequencing data sets, the total number of reads obtained is an arbitrary value set by the sequencing instrument and absolute abundance of each taxon is unknown (Gloor et al. 2017). Therefore, to account for the compositional nature of the data, we applied the centered log-ratio (CLR) transformation (Aitchison 1986). Because log transformation is only possible for positive values, we dealt with 0 count values by adding a pseudocount to the normalized read count for every taxon in every sample. Due to the genome size normalization, we set the pseudocount to the equivalent of one read divided by the average genome size for all taxa.

#### Statistical Analyses

We used the R package vegan (Oksanen et al. 2018) for diversity estimates. The Shannon index (Shannon 1948) was used to estimate alpha diversity, via the diversity function on the raw read count data (i.e., prior to genome size normalization and CLR transformation), because the calculation of the metric required positive integers. Differences between host species were investigated with an Akaike information criterion based stepwise regression to determine the best-fit general linear model, with surface decontamination, sequencing depth (number of unmapped reads per sample), and proportion of human reads per sample included as covariates. Beta diversity (a measure of interindividual variation) was investigated based on the presence/absence of microbial taxa as well as on the CLR abundance data, using Jaccard and Euclidean distances, respectively, calculated with the vegdist function. Ordination with NMDS was performed on the distance matrices with the metaMDS function in vegan. The NMDS stress value, which is a measure of the degree to which the distance between samples in the reduced dimensional space corresponds with the actual distance between samples (similar to a “goodness of fit” value), has been included in the figure legend of each NMDS plot. Permutational multivariate analysis of variance (PERMANOVA) was performed on the distance matrices with the adonis function in vegan. Host species, surface decontamination, number of unmapped reads per sample, and proportion of human reads per sample were included as covariates in the adonis model. To determine whether differences in within-group variation between host species were biasing inferences of a host-specific oral microbiome signature, a distance-based test for homogeneity of multivariate dispersions was performed with the vegan function betadisper. No such differences were detected, adding confidence to the PERMANOVA results.

To identify taxa which discriminated between host species, we carried out a random forest classification based on presence/absence data using the R package randomForest (Liaw and Wiener 2002) with 10,000 trees. This approach reports the out-of-bag estimated error (how often an individual was incorrectly assigned to a host species) and the variable importance (mean decrease in accuracy) of each taxon, which reflects the importance of the given taxon in determining the correct host species. For this analysis, the gorilla samples were excluded due to low sample size (*n* = 2).

We also investigated a subset of taxa unique to each host species and shared by >50% of samples from this species to determine whether they were likely oral taxa. As the two gorilla samples did not share any taxa at the species level, we investigated all of the species-level taxa unique to each of the two samples. We then compared these sets of taxa with the HOMD (Chen et al. 2010) and classified those present as “oral.” Taxa not present in the HOMD were manually classified as “oral” (based on the presence in the oral microbiome of nonhuman mammals), “host-associated” (present in nonoral mammalian microbiomes) or “not host-associated” through a literature search.

### Functional Analysis

#### Classification

The functional genic content of the microbial community in dental calculus was characterized by running the contamination-filtered reads through the HUMAnN2 pipeline (Franzosa et al. 2018), which identifies species-specific genes with the taxonomic profiler MetaPhlAn2 (Truong et al. 2015) and a built-in microbial pangenome database representing all known nonredundant protein-coding potential for each species identified by MetaPhlAn2, and more general functional characterization by alignment with DIAMOND (Buchfink et al. 2015) against the UniRef90 (Suzek et al. 2007) database. The mappings are weighted by quality and sequence length to estimate species-specific and total community gene family abundance. Metabolic pathways are also reconstructed based on genes annotated to metabolic enzymes in MetaCyc (Caspi et al. 2018), and the pathway abundance and coverage are reported.

#### Statistical Analyses

CLR normalization, NMDS ordination, and PERMANOVA were carried out for the pathway abundances as for the microbial analyses outlined above. PCA ordination was also carried out, using the prcomp R function (with data centered and scaled). Core pathways were defined as those containing >50% of the required enzymes in a sample (i.e., per sample pathway coverage >0.5) for the total community (i.e., not stratified by microbial species). Relative (proportional) abundance for specific pathways stratified by microbial species was also calculated by HUMAnN2 for each sample.

### AMR Profiles

#### Oral Bacteria

A list of oral bacteria (at genus and species level) was defined using species classified as “human_oral” by Mann et al. (2018) and supplemented by information gathered from the HOMD (Chen et al. 2010) and the dog and cat oral microbiota (Dewhirst et al. 2012, 2015). If the majority of identified species within a genus was associated with the oral cavity or upper respiratory tract, the entire genus was defined as oral; whereas if the genus contained both oral-associated species and environment-associated species, only oral-associated species were included. The list of oral taxa and additional details on classification are available in supplementary table S4, Supplementary Material online. Contamination-filtered fasta reads mapping to this list of oral taxa were extracted from Megan rma6 files with MaltExtract (Hübler et al. 2019) using the filter (-f) “full” and setting the --reads flag. The corresponding fastq reads were obtained using seqtk subseq (v1.2-r101, github.com/lh3/seqtk, accessed June 13, 2019) and reads for all extracted taxa were combined into one fastq file per sample.

#### Neisseria spp

All complete genome assemblies for *Neisseria* species (*n* = 183) available at NCBI Genome (accessed November 14, 2019) were downloaded and the fasta files concatenated into one file. Contamination-filtered fasta reads mapping to the *Neisseria* genus or any *Neisseria* species were extracted and the corresponding fastq reads obtained with MaltExtract and seqtk as above. These reads were then mapped to the *Neisseria* reference genomes with bwa mem v0.7.17 (Li and Durbin 2009; Li 2013) with the mismatch penalty (-B) set to 3 to allow mapping of reads with less similarity to the reference genomes that may be from related, currently uncharacterized species. All mapped reads were extracted with SAMTools v1.9 (samtools fastq --F4) and reads mapping to parts of the *Neisseria* reference assemblies forming plasmids were excluded, since plasmids can be exchanged between bacterial species through horizontal gene transfer, which can lead to erroneous taxonomic identification.

#### Blast against CARD

Reads from oral bacteria and *Neisseria* spp. were then aligned to the CARD v3.0.1 (modified February 19, 2019) (Jia et al. 2017), a curated collection of resistance determinant sequences, with blast v2.7.1+ (Altschul et al. 1990; Madden et al. 2009) using default parameters. The ARO accession number associated with each CARD sequence was used to obtain the AMR gene family of each sequence. Where reads matched multiple sequences in the CARD, the best hit was identified based on highest bit score. Where multiple hits had the same bit score, we compared the ARO terms and if all hits shared the same ARO information, we randomly chose one hit to carry forward. When ARO information was not identical, we manually identified a common higher level term of the hits: for example, all beta-lactamase families were combined into a single beta-lactamase group. We then calculated the number of reads per ARO accession per sample and normalized it by sample sequencing depth (number of extracted reads) for the oral bacteria analysis or by number of reads mapping to *Neisseria* for the targeted analysis. The abundance of ARO gene families for each sample was calculated by summing across the ARO accession abundances associated with each gene family.

#### Authentication

Authenticity was confirmed for a subset of the most abundant oral bacteria, including *Neisseria* spp. and *P. gingivalis*. The contamination-filtered fastq reads from these taxa extracted by MaltExtract were mapped to the relevant reference genomes (e.g., all 183 complete genomes for *Neisseria* and all 21 complete genomes for *P. gingivalis* downloaded from NCBI Genome, accessed November 14, 2019) with bwa mem v0.7.17 (Li and Durbin 2009; Li 2013) (-B 3). Alignments were filtered to remove unmapped reads with SAMTools v1.9 (samtools view -F4) and soft-clipped reads were removed with samclip (github.com/tseemann/samclip, accessed January 22, 2020, setting --max to 0). Misincorporation plots were generated with mapDamage v2.0.9 (Jónsson et al. 2013) with length (-l) set to 100 nt and --merge-reference-sequences to reduce disk usage.

### MAG Recovery

We attempted to recover draft MAGs from our final 12 samples following a similar strategy as described in Zhou et al. (2018) and Parks et al. (2017). Individual assembly per sample was performed by assembling reads into contigs with MEGAHIT v1.1.2 (Li et al. 2015) using default settings. Sample depth profiles were generated by mapping reads back to the contigs with BBMap v38.08 (Bushnell B, *BBMap*, sourceforge.net/projects/bbmap/, accessed 18 June, 2018). Contigs >1,500 bp in length were grouped into genome bins based on coverage covariance and tetramer frequencies using MetaBAT2 v2.12.1 (Kang et al. 2015) with default settings, where each bin theoretically represents the genome of a single strain. Assembly statistics and quality of each bin were assessed using the lineage-specific workflow in CheckM v1.0.12 (Parks et al. 2015). Medium-quality bins, or MAGs, were classified as those with an estimated ≥50% genome completeness and <10% strain contamination, as per the genome reporting standards for MAGs (Bowers et al. 2017). The taxonomic lineage of each medium-quality bin, as defined in the Genome Taxonomy Database (GTDB, release 89) (Parks et al. 2018), was determined with GTDB-Tk v1.1.1 (Chaumeil et al. 2019) (details in Supplementary Material online). For the eight highest quality draft MAGs (>90% completeness), misincorporation plots of reads mapped back to the binned contigs were generated using mapDamage v2.0.9 (Jónsson et al. 2013) as described above (i.e., -l 100 --merge-reference-sequences). To determine minimum sequencing depth required to recover high-quality MAGs (>90% completeness), we used seqtk sample to downsample each dental calculus sample to 15, 12.5, 10, 7.5, 5, and 2.5 million reads in triplicates (using a different random seed for each replicate) and repeated the MAG recovery and assessment steps as above. For each higher quality draft MAG, we determined the minimum sequencing depth at which the MAG was recovered at high quality and compared this depth with the percentage of sample reads aligned to the MAG (taken from the sample depth profiles generated with BBMap).

### Dietary Components Recovery

Counts of reads assigned by MALT to eukaryotic taxa were exported from MEGAN at the family and genus level and imported into R. Taxa with <10 assigned reads across all samples were excluded from further analysis. At the family level, assigned read counts were normalized across samples by adding a pseudocount of 0.1 and applying the CLR transformation. The proportion of specific genera per sample was calculated by taking genus level read counts and normalizing by sample sequencing depth.

### Host Genome Recovery

We collected published whole genome sequence data for seven reindeer (Li et al. 2017; Lin et al. 2019), one white-tailed deer (GCA_002102435.1), 47 gorillas (Prado-Martinez et al. 2013; Xue et al. 2015; Besenbacher et al. 2019), 24 brown bears, and three polar bears (Cahill et al. 2013, 2015; Benazzo et al. 2017; Barlow et al. 2018). Reads were mapped either merged (ancient samples) or paired-end (modern samples) to an outgroup reference genome assembly (white-tailed deer for reindeer: GCA_002102435.1, human for gorilla: GCA_000151905.3, and polar bear for brown bear: GCA_000687225.1) using bwa mem on default settings. Next, we excluded reads with a mapping quality score <30 and removed duplicate reads with SAMTools v0.1.19. Additionally, for each study host species, we mapped all reads to the mitochondrial references of white-tailed deer (NC_015247.1), polar bear (NC_003428.1), and western gorilla (NC_011120.1), respectively, following the same pipeline.

To investigate host genomic variation, we generated pseudohaploid sequences for each individual by randomly selecting a single high-quality base call (BaseQuality ≥30 and MapQuality ≥30) at each site covered by at least one read, excluding sites within repetitive regions (as identified from the repeatmask tracts) and for modern genomes sites with >2 times genome-wide coverage to minimize false bases from spurious mappings. A reference set of high-quality polymorphisms was made from all biallelic autosomal sites in the modern genomes and the reindeer museum sample Rt11, for which enough sequencing data were obtained to be included in the reference panel (supplementary table S7, Supplementary Material online). We then projected the low-coverage dental calculus samples onto the precalculated PCA space using the lsqproject function in smartpca (Patterson et al. 2006). Mitochondrial genome variation was investigated following the same pipeline but calling the majority allele at each covered site. We then used popart (Leigh and Bryant 2015) to create haplotype networks for all near-complete mitochondrial genomes (>90% complete). For the incomplete mitochondrial genomes, we calculated pairwise divergence to each of the modern genomes to obtain the most likely closest related haplotype. In addition, we also obtained de novo mitochondrial genomes using MITObim v1.9 (Hahn et al. 2013), a mitochondrial baiting, and iterative mapping method. The mitochondrial genomes of the western lowland gorilla (*Gorilla gorilla gorilla*, NC_011120.1), the brown bear (*U. arctos*, NC_003427.1), and the reindeer (*R. tarandus*, KM506758.1) were used as bait sequences for the de novo mitochondrial genome assembly of the eastern gorilla, brown bear, and reindeer samples, respectively. The merged host reads were used as input files for each sample. All mitochondrial genomes were annotated with the MITOS WebServer (Bernt et al. 2013) using default parameters.

## 

Raw sequencing data and binned contig assemblies of individual draft MAGs are archived at the European Nucleotide Archive under the project accession PRJEB33363 (sample-specific ENA accessions are provided in supplementary tables S1 and S5, Supplementary Material online). Sample metadata are provided in supplementary table S1, Supplementary Material online.

## Supplementary Material

msaa135_supplementary_data
