# Supplementary material for: Dental Calculus as a Tool to Study the Evolution of the Mammalian Oral Microbiome
Source: Mol Biol Evol. 2020 May 28;37(10):3003–22. doi: 10.1093/molbev/msaa135 (PMC7530607; doi:10.1093/molbev/msaa135)
Supplement: msaa135_supplementary_data [file msaa135_supplementary_data.zip › Brealey_et_al_dental_calculus_SuppMat_revised_20-05-18.pdf]

SUPPLEMENTARY MATERIAL

Dental calculus as a tool to study the evolution of the mammalian oral microbiome

Jaelle C. Brealey[1], Henrique G. Leitão[1], Tom van der Valk[1], Wenbo Xu[1], Katia Bougiouri[1], Love Dalén[2,3], Katerina Guschanski[1]

[1]Department of Ecology and Genetics, Animal Ecology, Evolutionary Biology Centre, Uppsala University, Norbyvägen 18D, 752 36, Uppsala, Sweden

[2]Department of Bioinformatics and Genetics, Swedish Museum of Natural History, 104 05, Stockholm, Sweden

[3]Centre for Palaeogenetics, Svante Arrhenius väg 20C, 106 91, Stockholm, Sweden

Correspondence:

Jaelle Brealey, Department of Ecology and Genetics, Animal Ecology, Evolutionary Biology Centre, Uppsala University, Norbyvägen 18D, 752 36, Uppsala, Sweden. Email: jaelle.brealey@ebc.uu.se

Katerina Guschanski, Department of Ecology and Genetics, Animal Ecology, Evolutionary Biology Centre, Uppsala University, Norbyvägen 18D, 752 36, Uppsala, Sweden. Email: katerina.guschanski@ebc.uu.se

Supplementary Methods

Decontamination tests

We investigated two types of surface decontamination techniques on dental calculus material immediately prior to DNA extraction. We took approximately 30 mg of each of the two gorilla dental calculus samples (collected from multiple healthy teeth within each individual) and split the material into three equal parts (11 ± 1 mg). We then compared the effects of exposing the calculus to UV light (245 nm) for 10 min, washing the calculus in 500 µl of 0.5M ethylenediaminetetraacetate (EDTA) for 30 seconds, and no surface decontamination as a control (Supplementary Fig. S3a). Based on real-time PCR of libraries prepared from these samples (see Materials and Methods: Library preparation and sequencing), we determined that neither decontamination treatment substantially reduced library quantity. We therefore continued with a surface decontamination procedure consisting of the UV light exposure followed by the EDTA wash (Ozga et al. 2016) for all subsequent calculus samples (3 bears and 2 reindeer; the other 3 bears and 3 reindeer were processed before the gorilla samples and did not undergo any surface decontamination).

NMDS ordination of microbial taxa abundances did not show marked differences among the six gorilla samples (Supplementary Fig. S2). SourceTracker analysis of microbial taxa abundances indicated that gorilla samples decontaminated with the EDTA wash contained the highest proportion of oral taxa (Supplementary Fig. S3b-c). These ETDA-washed gorilla samples were therefore retained for all subsequent analyses.

Contaminant identification: merged vs unmerged analysis

We compared the microbial taxa identified from the "merged reads" data (i.e. paired end reads that overlapped by >11 bp) with the "unmerged reads" data (i.e. paired end reads that did not overlap by >11 bp and could not be merged by AdapterRemoval) (Supplementary Fig. S5a). NMDS ordination based on presence/absence of taxa (calculated with Jaccard distance) showed some separation between merged and unmerged samples (Supplementary Fig. S5b), though this separation was not significant in a PERMANOVA (Supplementary Table S2). NMDS ordination based on CLR-normalised abundance of taxa (calculated with Euclidean distance) also showed some separation, with greater variation in the merged samples (Supplementary Fig. S5c), although again without significance (Supplementary Table S2).

We then directly compared the raw read counts of each taxon between the merged and unmerged reads on a per sample basis. The difference in read number between the unmerged and merged reads for each taxon in each sample was calculated and the distribution of all differences > 0 (i.e. more reads observed in the unmerged reads) was investigated (Supplementary Fig. S5d-e). Taxa

identified as outliers (defined as a difference > 1.5 interquartile ranges above the third quartile) in at least one sample were filtered out as putative contaminants (Supplementary Table S12). While it is possible that certain microbial structural features, e.g. the thick cell wall in Gram-positive bacteria, might bias preservation towards specific taxa and thereby affect our fragment length analysis (Key et al. 2017), a recent study of microbial DNA perseveration in human dental calculus found no association between cellular structures and microbial DNA fragmentation (Mann et al. 2018). Nonetheless, we confirmed that the majority of taxa in our putative contaminant list were non-host-associated environmental microorganisms through a literature search.

Post-mortem DNA damage investigations

Misincorporation plots were generated using mapDamage (Jónsson et al. 2013). During this investigation, we observed unusual damage patterns in several of our samples – rather than the expected incremental rise in deamination towards read ends, several samples showed a drop in the frequency of C-to-T substitutions from the penultimate base to the terminal base (e.g. comparison of damage patterns in Ua9 MAGs compared to Ua14 in Supplementary Fig. S11). It has been suggested previously that barcodes with a terminal G or C have reduced ligation efficiency to damaged cytosines at 5' ends of reads (Rohland et al. 2015). We therefore examined damage profiles for 14 most abundant bacterial taxa in the post-filtering dataset (Supplementary Table S6), using mapDamage as part of the EAGER pipeline v1.92.37 (Peltzer et al. 2016). We only considered cases with more than 10000 reads mapping to a taxon reference genome in a given sample in order to have enough coverage to discern a clear pattern above the background noise. We found that samples containing barcodes with a terminal G or C were more likely to show the unusual pattern ($\chi^2 = 31.11$, df = 1, p-value < 0.0001) (Supplementary Table S13).

Metagenome-assembled genome (MAG) taxonomic identification

The taxonomic lineage of each draft MAG was determined with the Genome Taxonomy Database Toolkit (GTDB-Tk) (Chaumeil et al. 2019) using the 'classify_wf' workflow with default settings. This workflow classifies bacterial genomes by placing them into concatenated protein reference trees based on a set of 120 bacterial marker genes and the GTDB reference tree (Parks et al. 2018). The GTDB phylogeny is constructed from RefSeq and Genbank genomes that include both cultured microorganism assemblies and draft genomes of uncultured microorganisms generated from metagenomic and single-cell sequencing (Parks et al. 2018). As described in (Chaumeil et al. 2019), in the classify_wf workflow, first genes are called using Prodigal (Hyatt et al. 2010) and the 120 bacterial marker genes are identified using HMM models with HMMER (Eddy 2011). The marker genes are aligned to their respective HMM model and the multiple sequence alignments concatenated and trimmed to approximately 5000 amino acids. This alignment is then used to

determine the maximum-likelihood placement of each MAG in the GTDB-Tk reference tree, using pplacer (Matsen et al. 2010). The taxonomic lineage of each MAG is then determined by its placement in this reference tree. Where rank assignments from the topology of the reference tree are ambiguous, taxonomy is resolved using relative evolutionary divergence. Average nucleotide identity (ANI), as calculated with FastANI (Jain et al. 2018) is used to establish species assignments within a genus.

Supplementary References

Chaumeil P-A, Mussig AJ, Hugenholtz P, Parks DH. 2019. GTDB-Tk: a toolkit to classify genomes with the Genome Taxonomy Database. Bioinformatics. 36:1925–1927.

Eddy SR. 2011. Accelerated profile HMM searches. PLoS Comput Biol. 7:e1002195.

Hyatt D, Chen GL, LoCascio PF, Land ML, Larimer FW, Hauser LJ. 2010. Prodigal: Prokaryotic gene recognition and translation initiation site identification. BMC Bioinformatics. 11:119.

Jain C, Rodriguez-R LM, Phillippy AM, Konstantinidis KT, Aluru S. 2018. High throughput ANI analysis of 90K prokaryotic genomes reveals clear species boundaries. Nat Commun. 9:5114.

Jónsson H, Ginolhac A, Schubert M, Johnson PLF, Orlando L. 2013. MapDamage2.0: Fast approximate Bayesian estimates of ancient DNA damage parameters. Bioinformatics. 29:1682–1684.

Key FM, Posth C, Krause J, Herbig A, Bos KI. 2017. Mining Metagenomic Data Sets for Ancient DNA: Recommended Protocols for Authentication. Trends Genet. 33:508–520.

Mann AE, Sabin S, Ziesemer K, Vågene ÅJ, Schroeder H, Ozga AT, Sankaranarayanan K, Hofman CA, Fellows Yates JA, Salazar-García DC, et al. 2018. Differential preservation of endogenous human and microbial DNA in dental calculus and dentin. Sci Rep. 8:9822.

Matsen FA, Kodner RB, Armbrust EV. 2010. pplacer: linear time maximum-likelihood and Bayesian phylogenetic placement of sequences onto a fixed reference tree. BMC Bioinformatics. 11:538.

Ozga AT, Nieves-Colón MA, Honap TP, Sankaranarayanan K, Hofman CA, Milner GR, Lewis CM, Stone AC, Warinner C. 2016. Successful enrichment and recovery of whole mitochondrial genomes from ancient human dental calculus. Am J Phys Anthropol. 160:220–228.

Parks DH, Waite DW, Skarshewski A, Chuvochina M, Rinke C, Hugenholtz P, Chaumeil P-A. 2018. A standardized bacterial taxonomy based on genome phylogeny substantially revises the tree of life. Nat Biotechnol. 36:996–1004.

Peltzer A, Jäger G, Herbig A, Seitz A, Kniep C, Krause J, Nieselt K. 2016. EAGER: efficient ancient genome reconstruction. Genome Biol. 17:60.

Rohland N, Harney E, Mallick S, Nordenfelt S, Reich D. 2015. Partial uracil-DNA-glycosylase treatment for screening of ancient DNA. Philos Trans R Soc B Biol Sci. 370:20130624.

Supplementary Figures

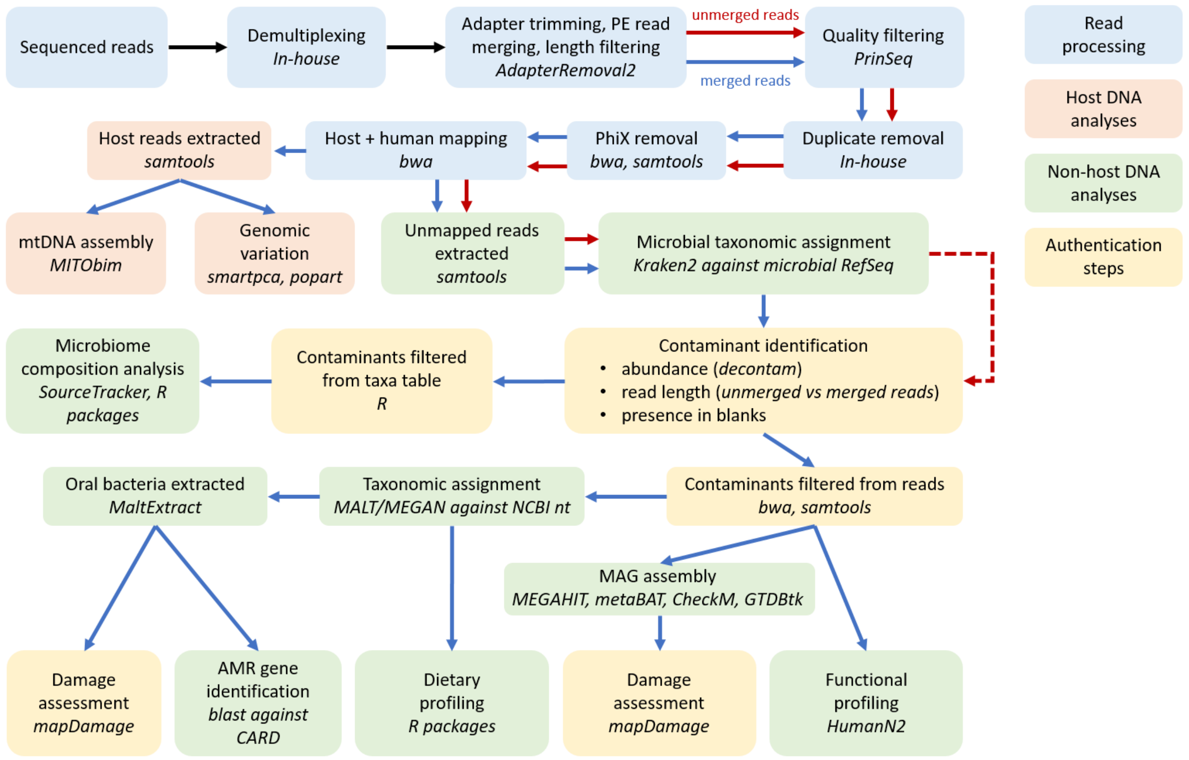

Supplementary Fig. S1. Summary of steps in data processing, contamination identification and subsequent analyses. Key tools used at each step are included in *italics*. During the adapter trimming step, sequenced paired end reads (black arrows) that overlapped were merged and used for all subsequent analyses (blue arrows). Paired end reads that could not be merged were assumed to derive from modern contaminant taxa with longer DNA fragments. The forward read of these unmerged reads (solid red arrows) were taken through the data processing and microbial taxonomic assignment steps and used to identify putative contaminating taxa (dashed red arrow). They were not used for any subsequent analyses.

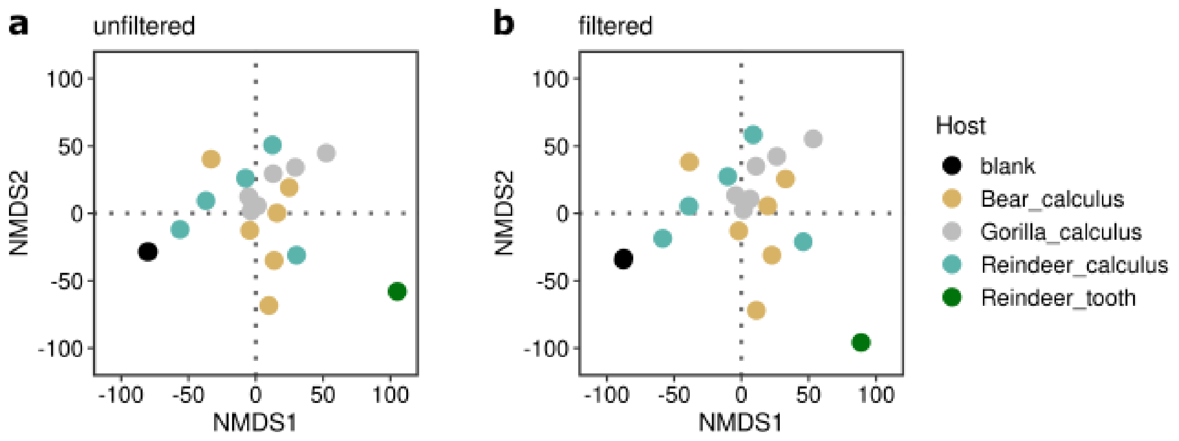

Supplementary Fig. S2. Beta diversity of all samples before **(a)** and after **(b)** filtering out putative contaminants. NMDS calculated using Euclidean distance of CLR-normalised abundance of microbial taxa. Samples are coloured by type (blank negative controls, calculus from each of the three host species or tooth sample from a reindeer specimen). In **(b)** the blank samples have not been filtered. NMDS stress on **(a)**: 0.1302; NMDS stress on **(b)**: 0.1303.

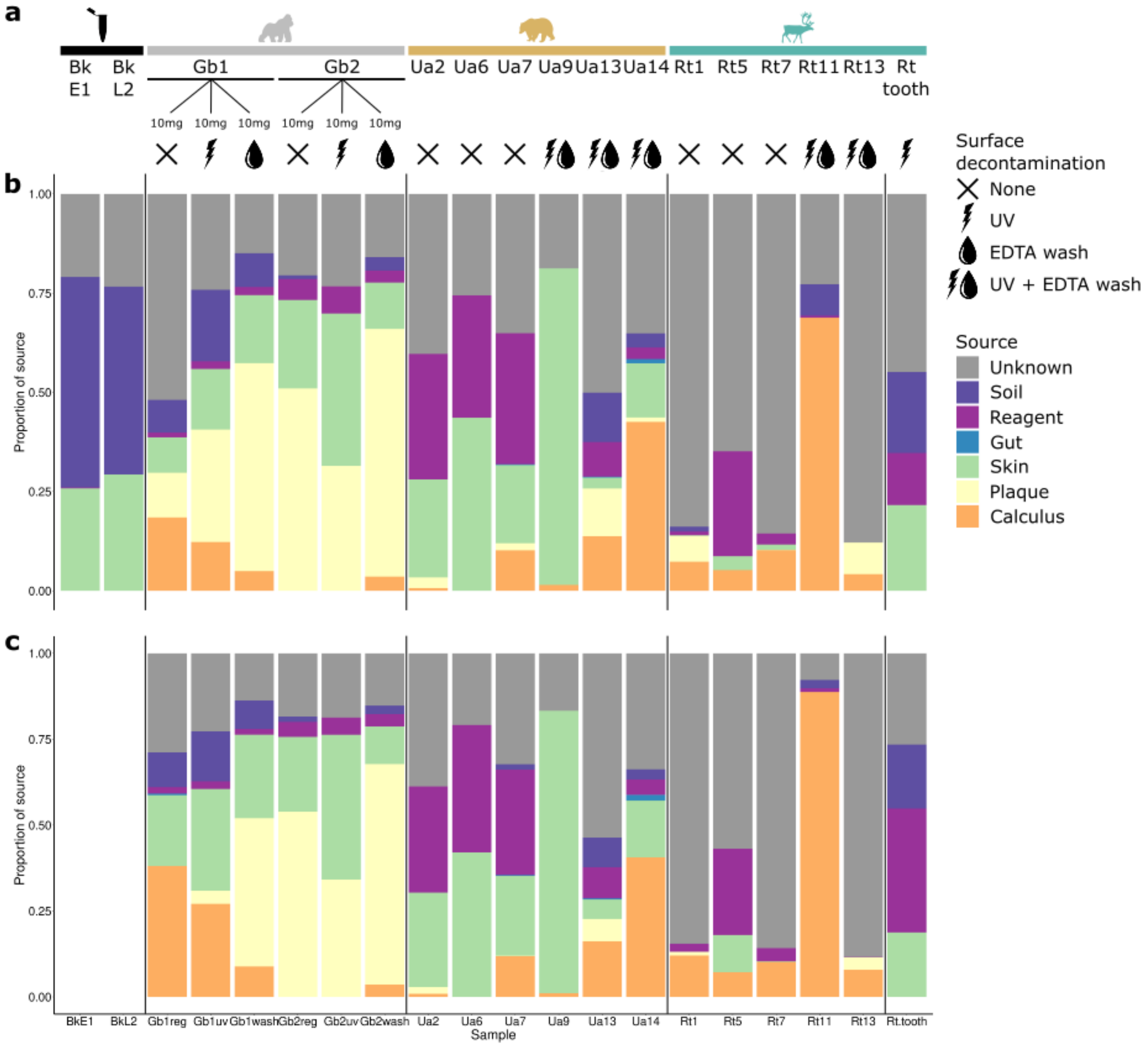

Supplementary Fig. S3. Proportions of source contributions to the microbial communities of samples with different surface decontamination treatments before and after bioinformatic filtering of contaminant taxa. **a)** Schematic of laboratory surface decontamination treatment procedures before DNA extraction. Surface decontamination treatment was tested on two gorilla calculus samples by splitting each into three, one which had no decontamination treatment, one which was exposed to UV light (245 nm) for 10 min and one which was washed in 500 µl of 0.5M EDTA for 30 seconds. Surface decontamination treatment was not performed on a subset of bear and reindeer calculus samples, while the remaining were treated first with the UV light exposure followed by the EDTA wash, as indicated. The reindeer tooth fragment was exposed to UV light (245 nm) for 10 min on each side before being ground into a powder for DNA extraction. **b)** Proportions of source contributions to the microbial communities (identified taxonomically at the species level) contained in the blank negative controls (BkE1 and BkL2), the dental calculus samples and one reindeer tooth sample before bioinformatic filtering of contaminant taxa. **c)** Source proportions after bioinformatic filtering of contaminant taxa. Proportions were estimated by SourceTracker, using modern human

oral, human skin, human gut, laboratory reagent, and soil microbiome datasets as sources. Contaminant taxa were identified using fragment length, presence in blanks and relative abundance (see Supplementary Fig. S1 and Supplementary Methods for the procedure used to identify contaminants). Note the proportional reduction of the “soil” source in the samples after filtering.

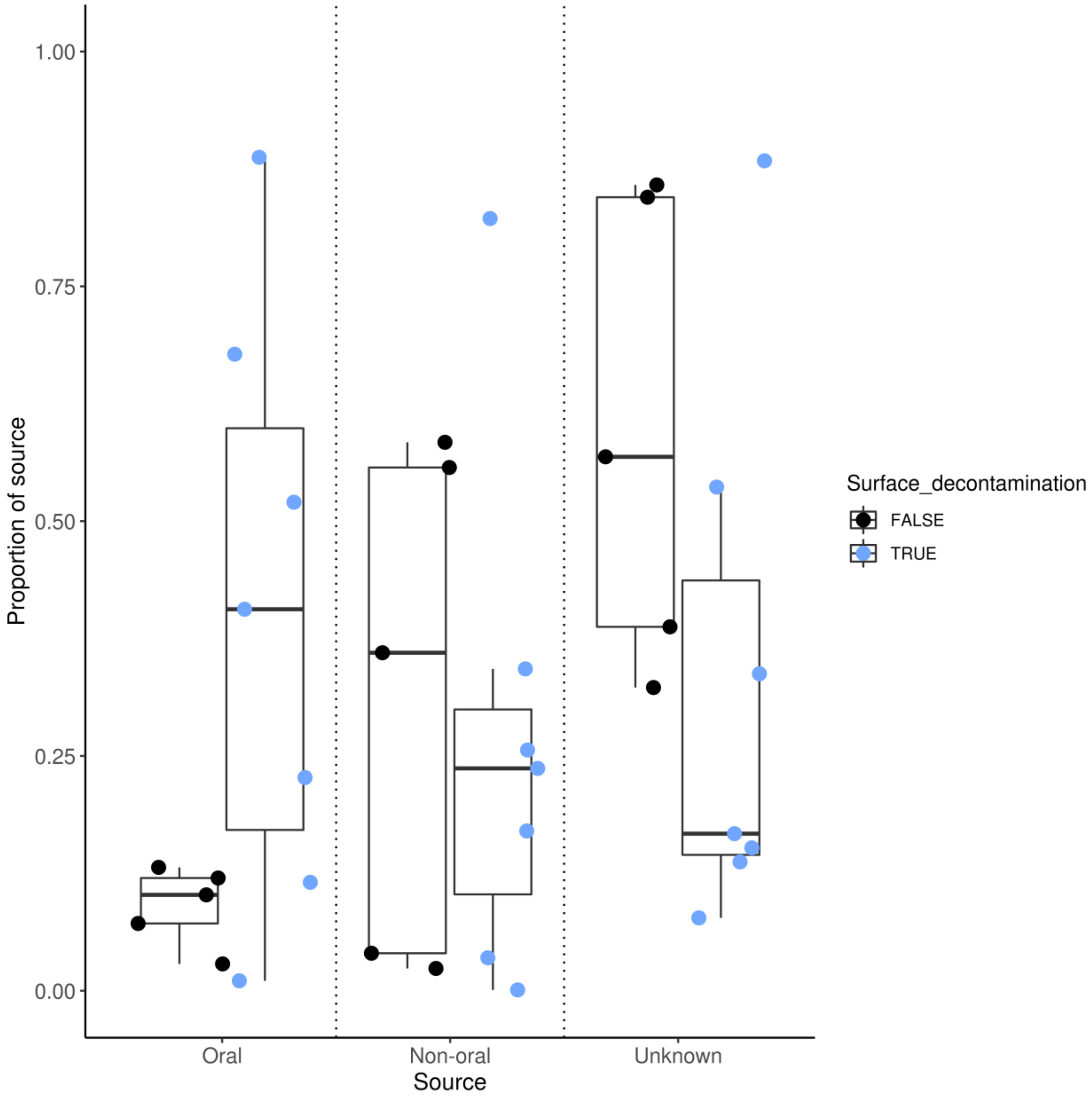

Supplementary Fig. S4. Proportions (visualised as Tukey boxplots) of microbial taxa assigned by SourceTracker to oral, non-oral and unknown sources in dental calculus samples of all studied host species that were surface decontaminated before DNA extraction and those that were extracted without surface decontamination. Surface decontamination consisted of a 10 min UV exposure and/or a 30 sec wash in 0.5M EDTA. Oral taxa include those assigned to human historical dental calculus and modern dental plaque microbiomes; non-oral taxa include those assigned to human skin, human gut, soil and laboratory microbiome sources; unknown taxa are those that were not assigned to any of the provided sources.

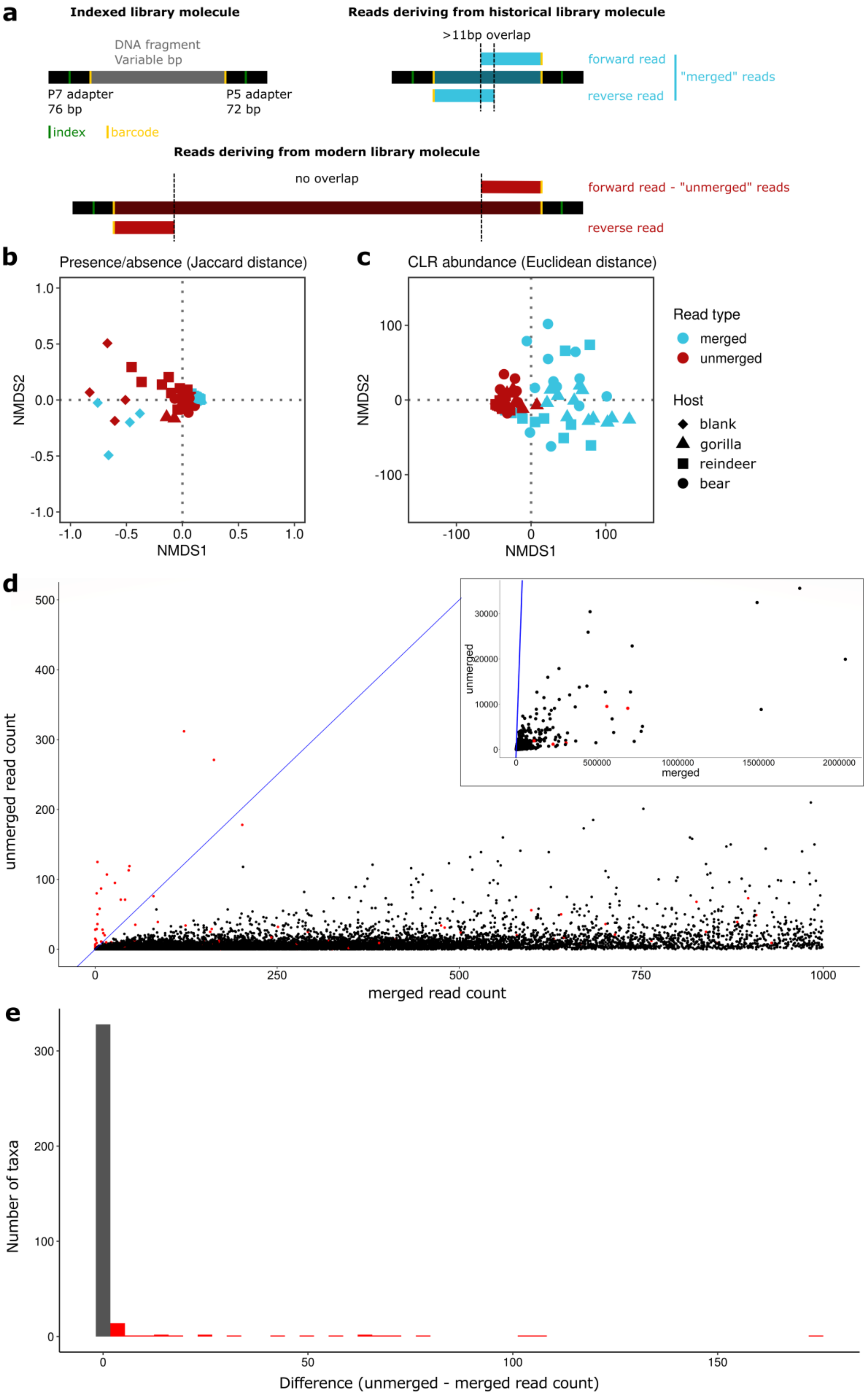
a
Indexed library molecule
DNA fragment
Variable bp
P7 adapter
76 bp
P5 adapter
72 bp
index
barcode
Reads deriving from historical library molecule
>11bp overlap
forward read
reverse read
"merged" reads
Reads deriving from modern library molecule
no overlap
forward read - "unmerged" reads
reverse read
b
Presence/absence (Jaccard distance)
NMDS1
NMDS2
c
CLR abundance (Euclidean distance)
NMDS1
NMDS2
Read type
merged
unmerged
Host
blank
gorilla
reindeer
bear
d
unmerged read count
merged read count
unmerged
merged
e
Number of taxa
Difference (unmerged - merged read count)

Supplementary Fig. S5. Identifying potential contaminant taxa from comparison of microbial taxa present in merged and unmerged reads from dental calculus and blank negative control samples. **a)** Schematic of an indexed library molecule, showing the adapter sequences in black, the Illumina index sequences in green and the inline barcodes in yellow ligated to the DNA fragment (grey). Paired-end reads sequenced from historical DNA fragments (blue) are expected to derive from short library molecules and can therefore be "merged", while read pairs sequenced from modern DNA fragments (red) are expected to derive from long libraries molecules, do not overlap and therefore remain "unmerged". We required > 11bp overlap for merging the paired-end reads. The forward reads of unmerged pairs were investigated further as deriving from potential modern contaminating taxa. **b)** NMDS on Jaccard distances from presence/absence of microbial taxa and **c)** NMDS on Euclidian distances from CLR-normalised abundance of microbial taxa in merged (blue) and unmerged (red) datasets. Host species of each sample (or negative blank control) is indicated by shape. NMDS stress on **(b)**: 0.085; NMDS stress on **(c)**: 0.106. **d)** Comparison of raw read counts between merged and unmerged reads, where each point represents a single taxon in a single sample. Taxa above the blue parity line (y=x) were more abundant in the unmerged reads in at least one sample, and were investigated further as possible modern contaminants. These potential contaminants are highlighted in red for all samples, including those in which a possible contaminant fell below the parity line. Note that this is a zoomed-in plot to allow visualisation of the pattern, the full plot is shown in the inset, where it is clear that the vast majority of taxa within all samples are more abundant in the merged reads. **e)** Distribution of the differences in read number, calculated as unmerged minus merged reads, only shown for differences > 0 (i.e. those falling above the blue parity line in **(d)**). Taxa identified as outliers (defined as a difference > 1.5 interquartile ranges above the third quartile), shown in red. These were filtered out as putative contaminants if observed in at least one sample.

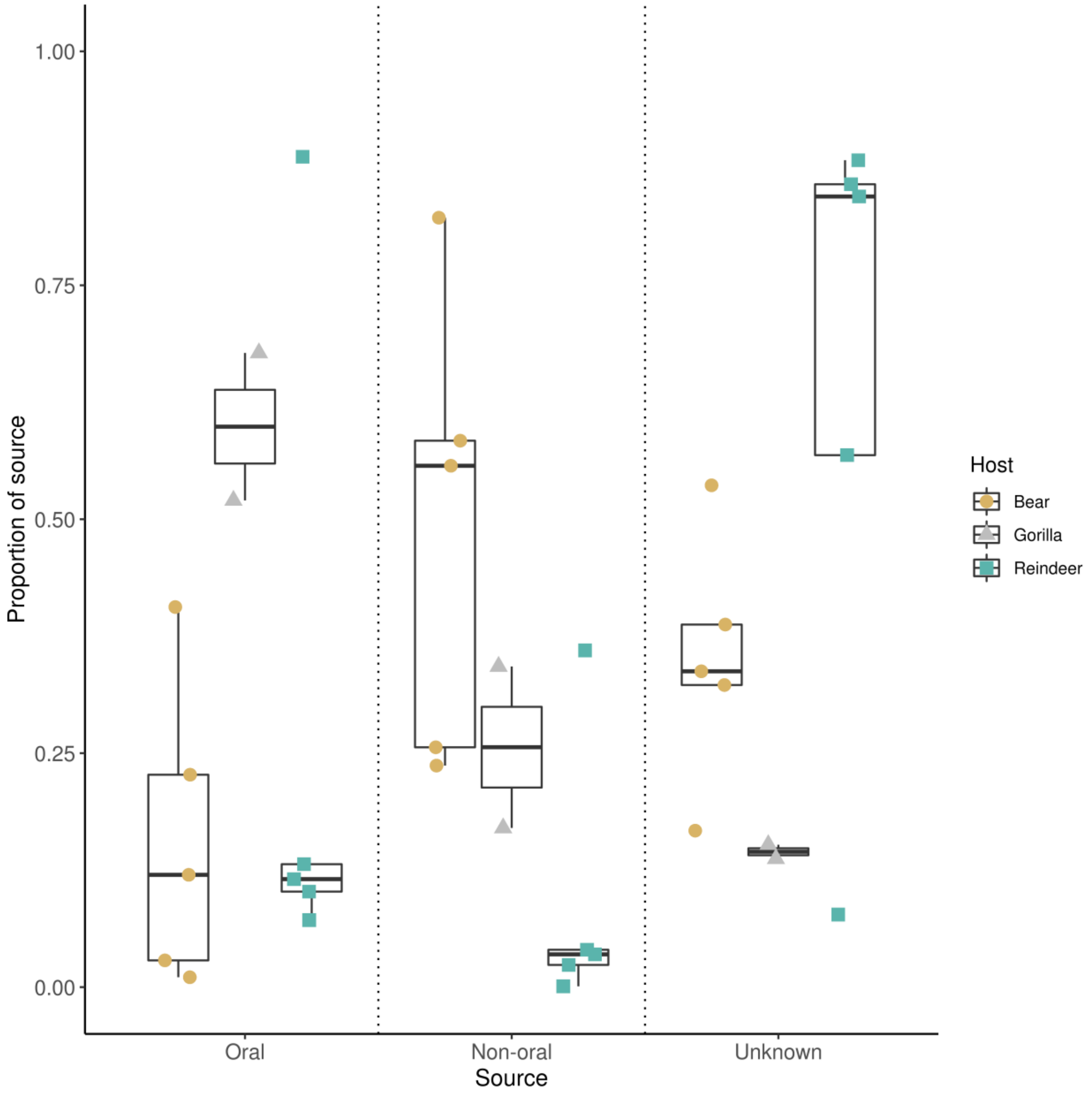

Supplementary Fig. S6. Proportions (visualised as Tukey boxplots) of microbial taxa assigned by SourceTracker to oral, non-oral and unknown sources in bear, gorilla and reindeer dental calculus samples. Oral taxa include those assigned to human historical dental calculus and modern dental plaque microbiomes; non-oral taxa include those assigned to human skin, human gut, soil and laboratory microbiome sources; unknown taxa are those that were not assigned to any of the provided sources.

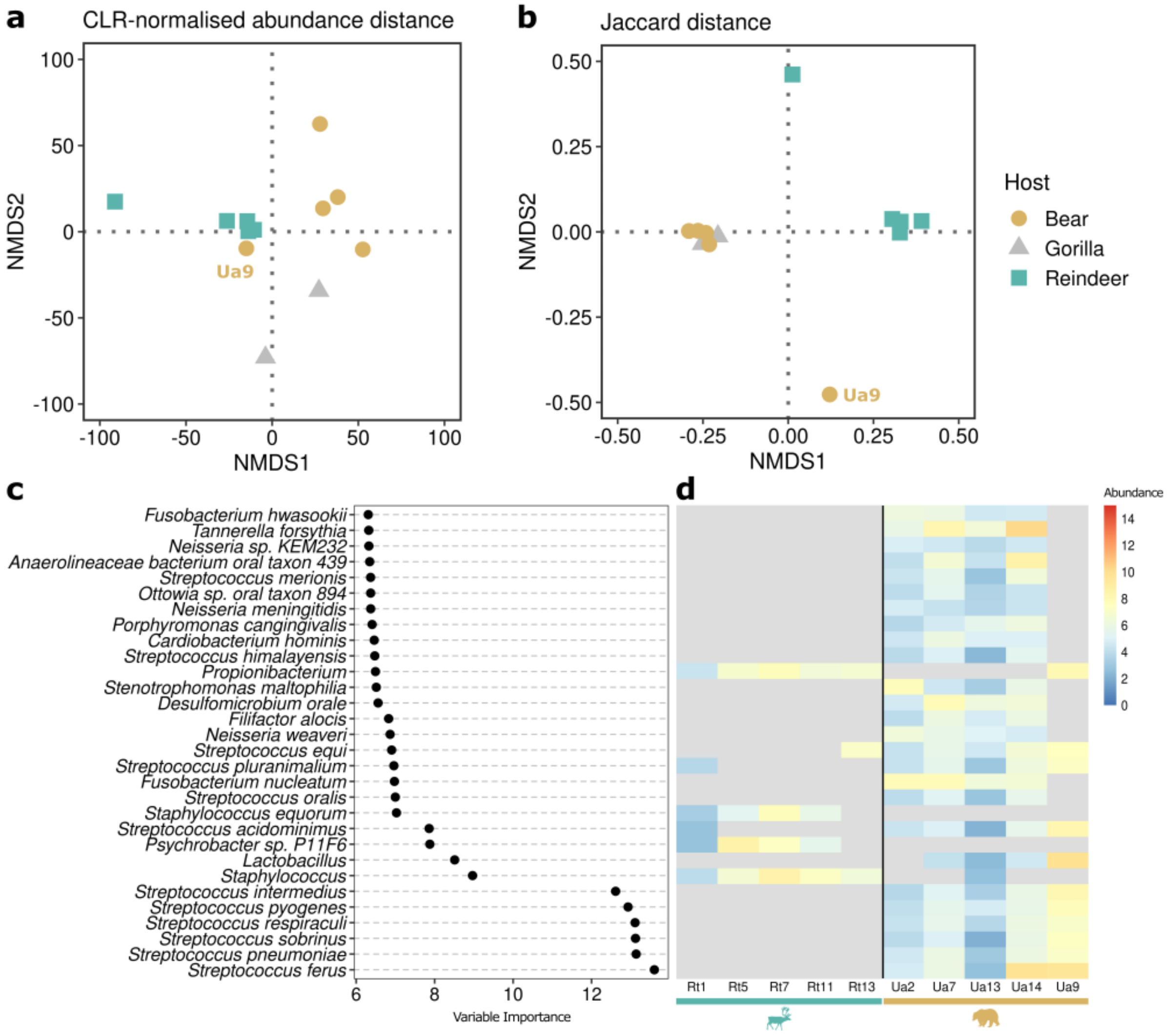

Supplementary Fig. S7. Beta diversity of all dental calculus samples, including the caries bear Ua9, after contaminant-filtering. **a)** NMDS calculated using Euclidean distance of CLR-normalised abundance of microbial taxa. **b)** NMDS calculated using Jaccard distance of presence/absence of microbial taxa. Samples are coloured by host species. NMDS stress on **(a)**: 0.060; NMDS stress on **(b)**: 0.113. **c)** Random forest variable importance plot of the 30 most discriminatory taxa comparing bear (including caries bear Ua9) and reindeer samples, based on presence/absence data after contamination filtering. The correct host species could be assigned in 90.0% of cases. **d)** CLR normalised abundance of the top 30 taxa in **(c)** in the bear and reindeer samples. Taxa that were not detected in a sample are coloured grey.

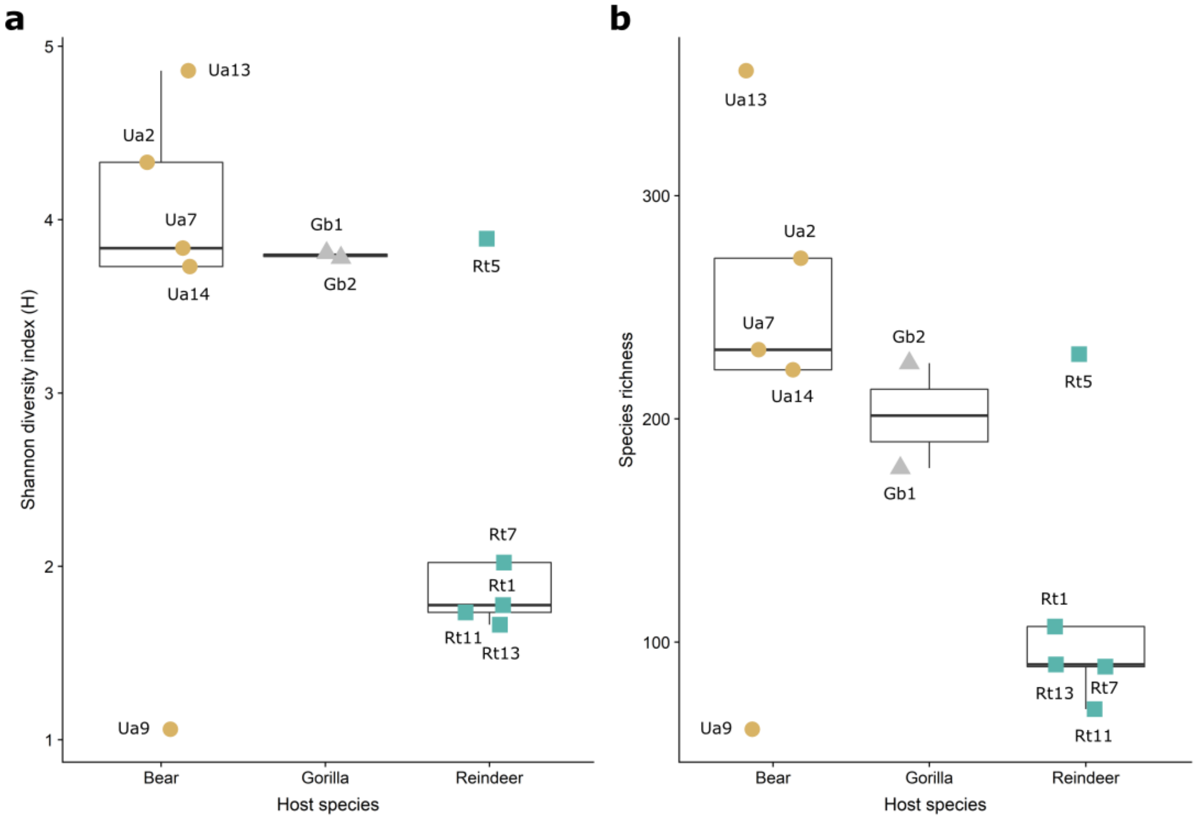

Supplementary Fig. S8. Alpha diversity (visualised as Tukey boxplots) in dental calculus samples from the three host species measured by **(a)** Shannon diversity index (takes into account both presence and abundance of microbial taxa) and **(b)** species richness (number of microbial taxa present). Points are labelled by their sample ID. The caries bear Ua9 has particularly low alpha diversity compared to healthy bear samples. Reindeer show overall lower diversity values compared to bear and gorilla samples.

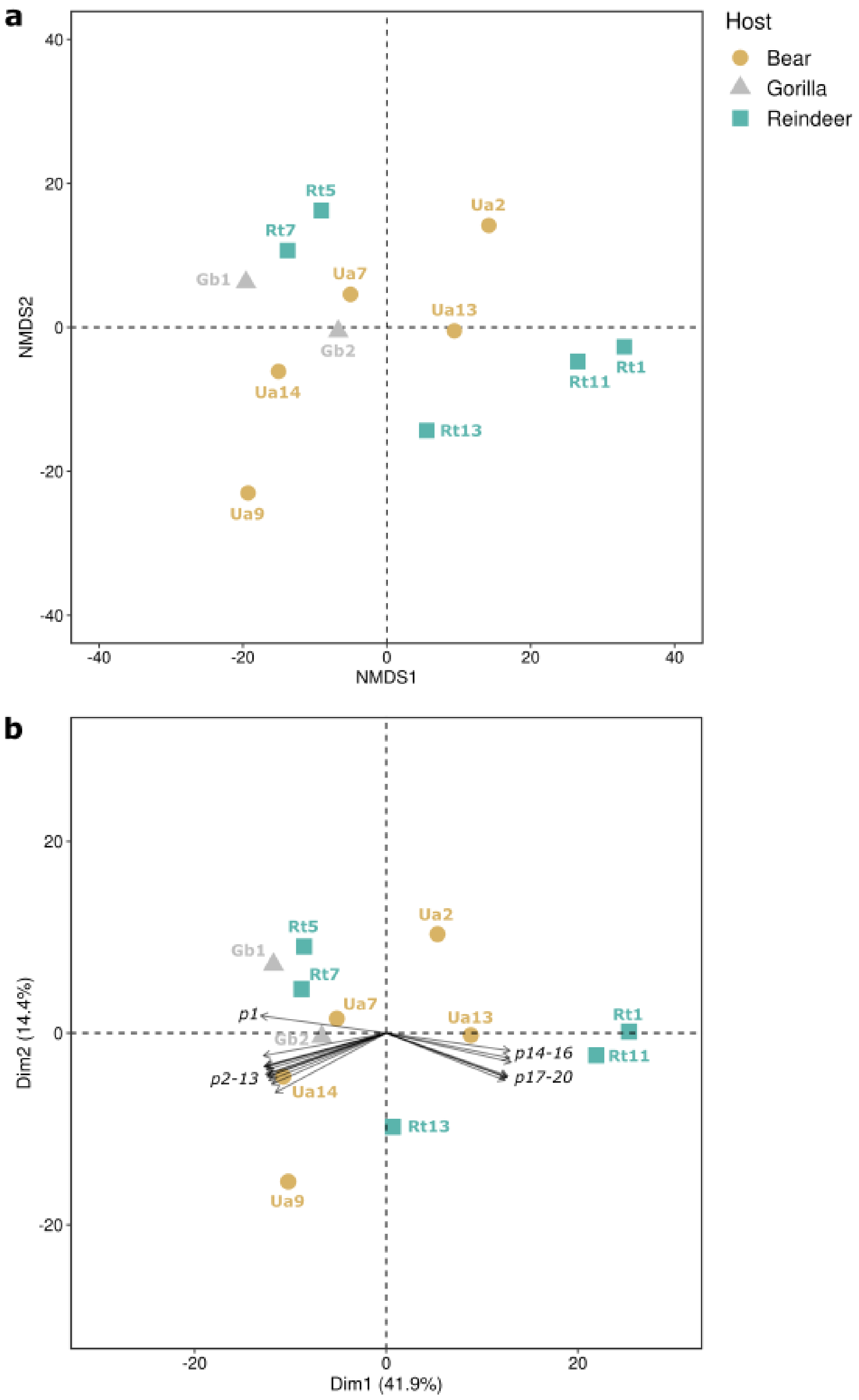

Supplementary Fig. S9. Beta diversity based on the CLR normalised metabolic pathways abundance data from the HUMAnN2 functional analysis. **a)** NMDS and **b)** PCA using a distance matrix based on the Euclidean distances. Points are labelled with sample ID and are coloured by host species. NMDS

stress in **(a)**: 0.110. In **(b)** arrows indicate the top 20 pathways contributing to sample separation along the first two components. Pathway (p) 1-13 are associated with core biosynthesis and central metabolism functions and p14-20 are generally associated with bacterial metabolism, particularly degradation of carbon and plant metabolites. Pathways:

p1	PWY0-162	superpathway of pyrimidine ribonucleotides de novo biosynthesis
p2	PWY-5686	UMP biosynthesis I
p3	ARO-PWY	chorismate biosynthesis I
p4	COMPLETE-ARO-PWY	superpathway of aromatic amino acid biosynthesis
p5	PWY-2942	L-lysine biosynthesis III
p6	PWY-724	superpathway of L-lysine, L-threonine and L-methionine biosynthesis II
p7	PWY-6121	5-aminoimidazole ribonucleotide biosynthesis I
p8	PWY-6122	5-aminoimidazole ribonucleotide biosynthesis II
p9	PWY-6277	superpathway of 5-aminoimidazole ribonucleotide biosynthesis
p10	PWY-841	superpathway of purine nucleotides de novo biosynthesis I
p11	THRESYN-PWY	superpathway of L-threonine biosynthesis
p12	PWY-3001	superpathway of L-isoleucine biosynthesis I
p13	ANAGLYCOLYSIS-PWY	glycolysis III (from glucose)
p14	PWY-5181	toluene degradation III (aerobic) (via p-cresol)
p15	PWY-6185	4-methylcatechol degradation (ortho cleavage)
p16	PWY-7323	superpathway of GDP-mannose-derived O-antigen building blocks biosynthesis
p17	PWY-6182	superpathway of salicylate degradation
p18	PWY-5431	aromatic compounds degradation via β-ketoadipate
p19	PWY-5417	catechol degradation III (ortho-cleavage pathway)
p20	CATECHOL-ORTHO-CLEAVAGE-PWY	catechol degradation to β-ketoadipate

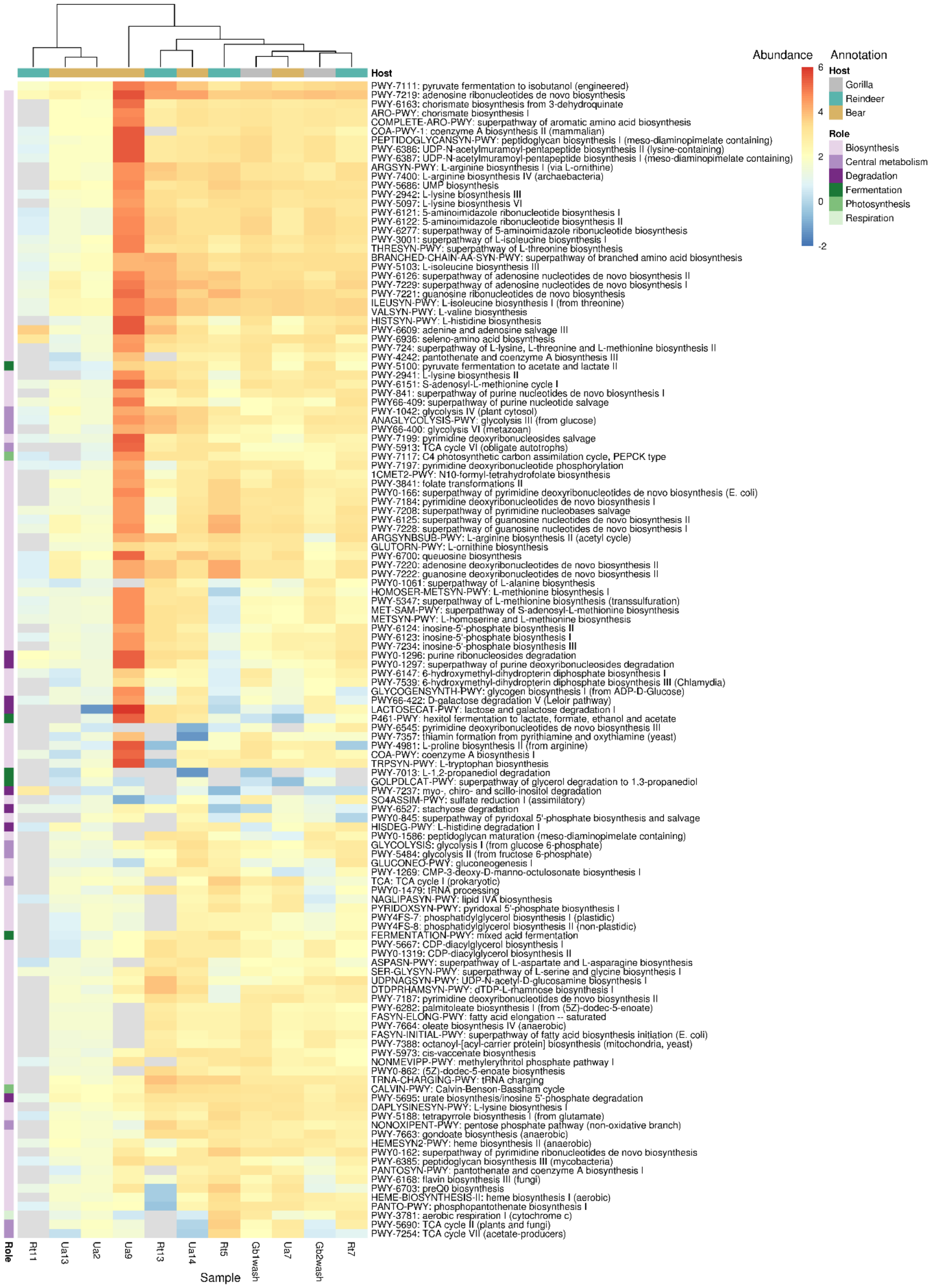

Supplementary Fig. S10. Core functions are shared by the oral microbiome of gorillas, bears and reindeer. CLR normalised abundance of MetaCyc metabolic pathways containing >50% of required enzymes, as identified in by HUMAnN2 in each dental calculus sample. Taxa that were not detected in a sample are coloured grey. Metabolic pathways are annotated with their main role in metabolism. Samples and pathways are hierarchically clustered by dissimilarity implemented by pheatmap.

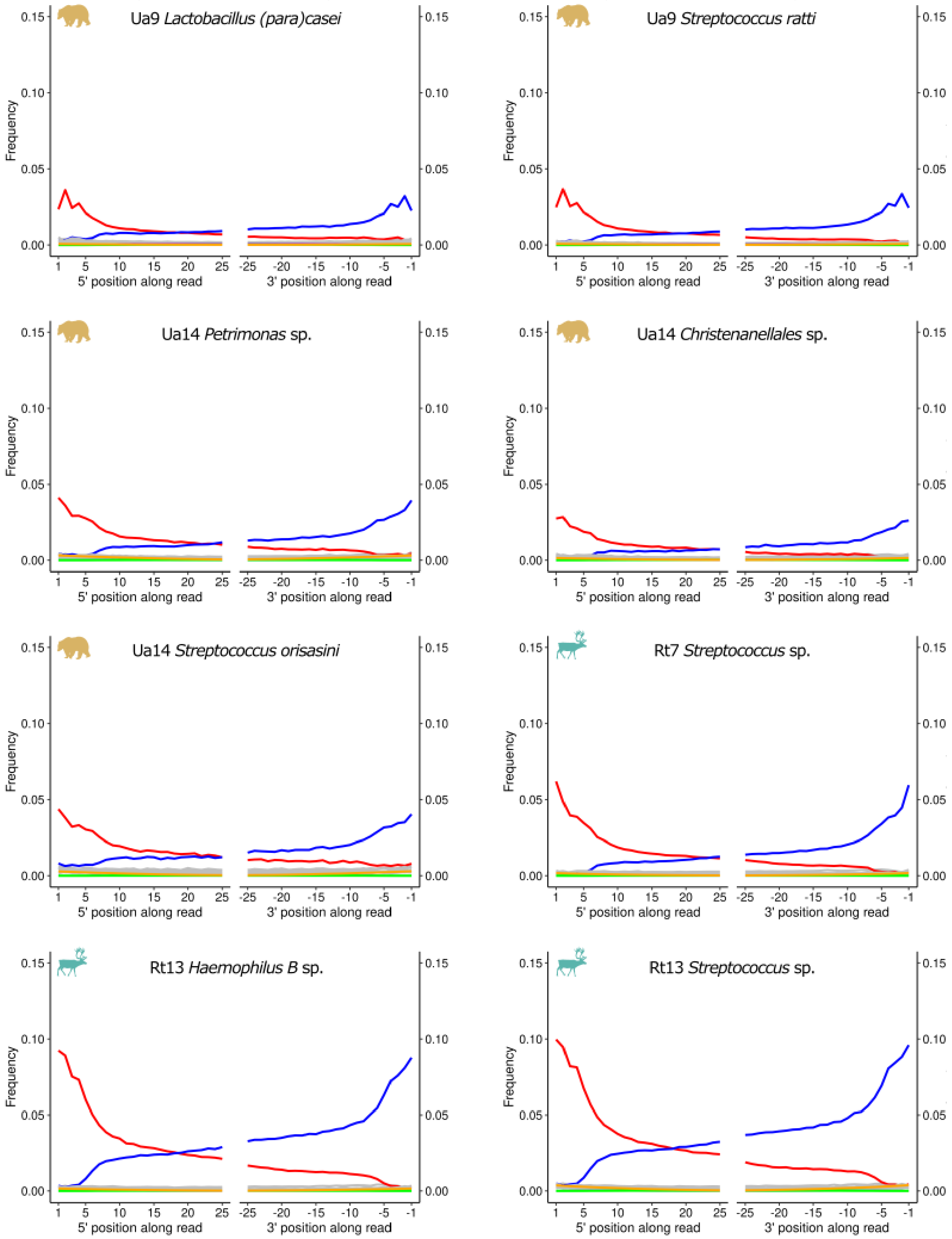

Supplementary Fig. S11. Highest quality draft MAGs (> 90% completeness) recovered from dental calculus samples show deamination patterns consistent with post-mortem DNA damage. Sample reads were mapped to the corresponding MAG assembly and the substitution frequencies determined with mapDamage. Frequency of C-to-T substitutions in reads compared to the reference are shown in red and G-to-A substitutions in blue. All other substitutions are shown in grey,

insertions in purple, deletions in green and soft-clipping in orange, representing background noise. Each draft MAG is labelled with the sample ID it was recovered from and the taxonomy it was assigned by GTDB-Tk. The apparent decrease in deamination frequency at the terminal base of reads from Ua9 is probably due to a technical bias during library preparation (see Supplementary Methods and Supplementary Table S6).

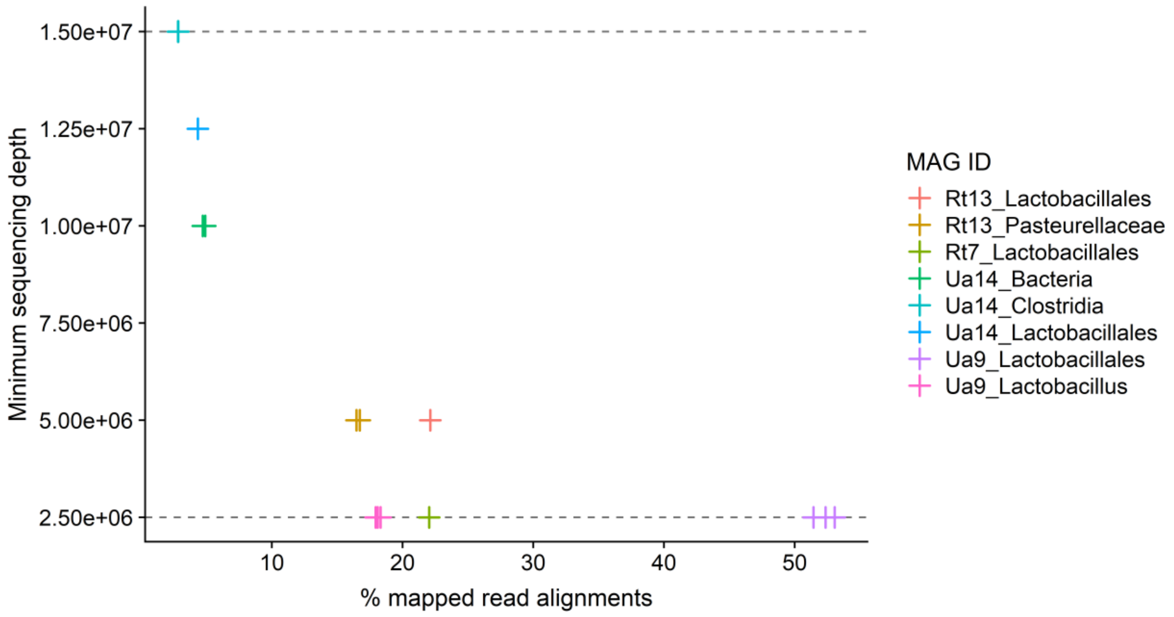

Supplementary Fig. S12. Relationship between the sequencing depth required to recover draft MAGs from historical dental calculus samples and their estimated abundance in the microbial community. Each of the highest quality draft MAGs recovered from the dental calculus samples (defined as MAGs with > 90% genome completeness and < 10% strain contamination, as estimated by CheckM) was assigned a MAG ID (sampleID_lineage). Each sample was then downsampled in triplicate to 15, 12.5, 10, 7.5, 5 and 2.5 million reads and MAG recovery and assessment repeated. The lowest sequencing depth at which each MAG was recovered at high quality is plotted against the percentage of read alignments to the assembled MAG, as an estimate of the abundance of the MAG in the sample. As expected, MAGs at higher abundance in a sample (e.g. Ua9_Lactobacillales) required fewer sequencing reads to be recovered at high quality. Dotted lines mark the upper and lower bounds of the downsampling.

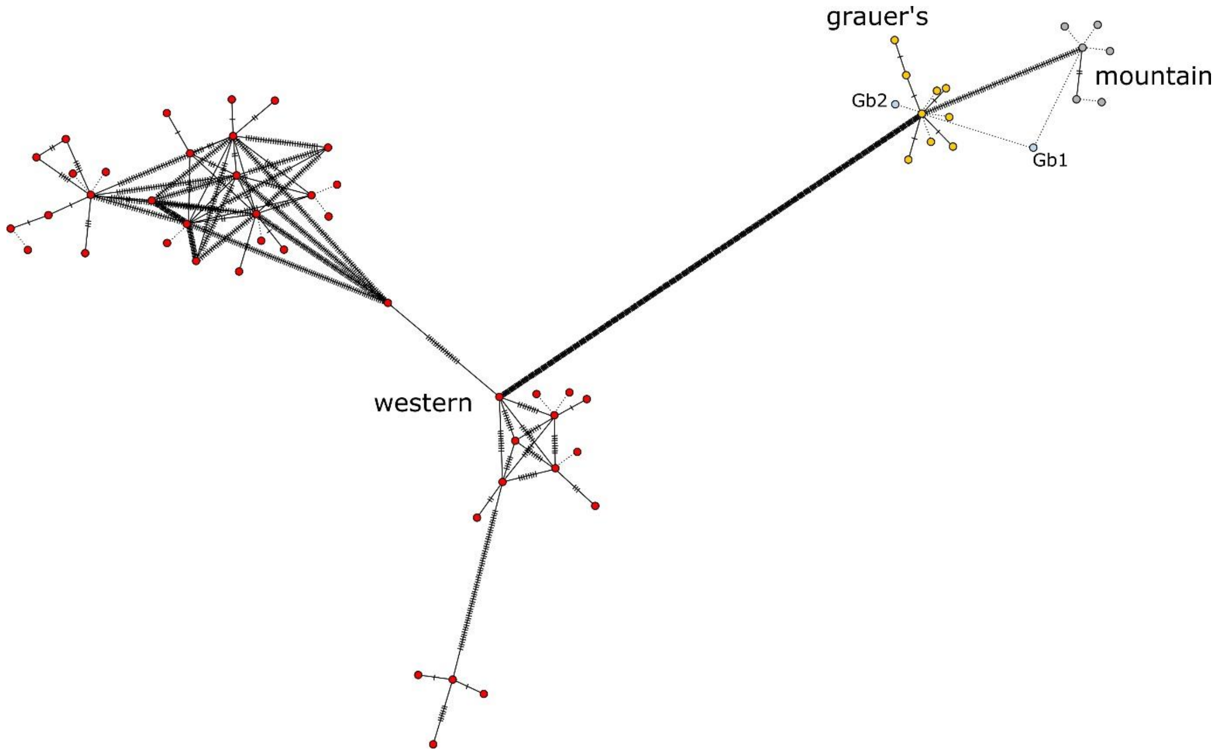

Supplementary Fig. S13. MtDNA haplotype network for gorillas. Each dot represents a sample, with ticks on the connecting lines showing the number of base pair substitutions between the haplotypes. Dotted lines represent identical haplotypes or in the case of dental calculus samples (in light blue), the predicted most closely related haplotype. Gb2 clusters with the Grauer's gorillas, consistent with its museum-recorded subspecies designation. Gb1, a museum-designated mountain gorilla, groups with the Eastern gorillas but based on solely mtDNA cannot be identified at the subspecies level. However, using whole genome data, Gb1 clusters more closely to mountain gorillas (Fig. 5b).

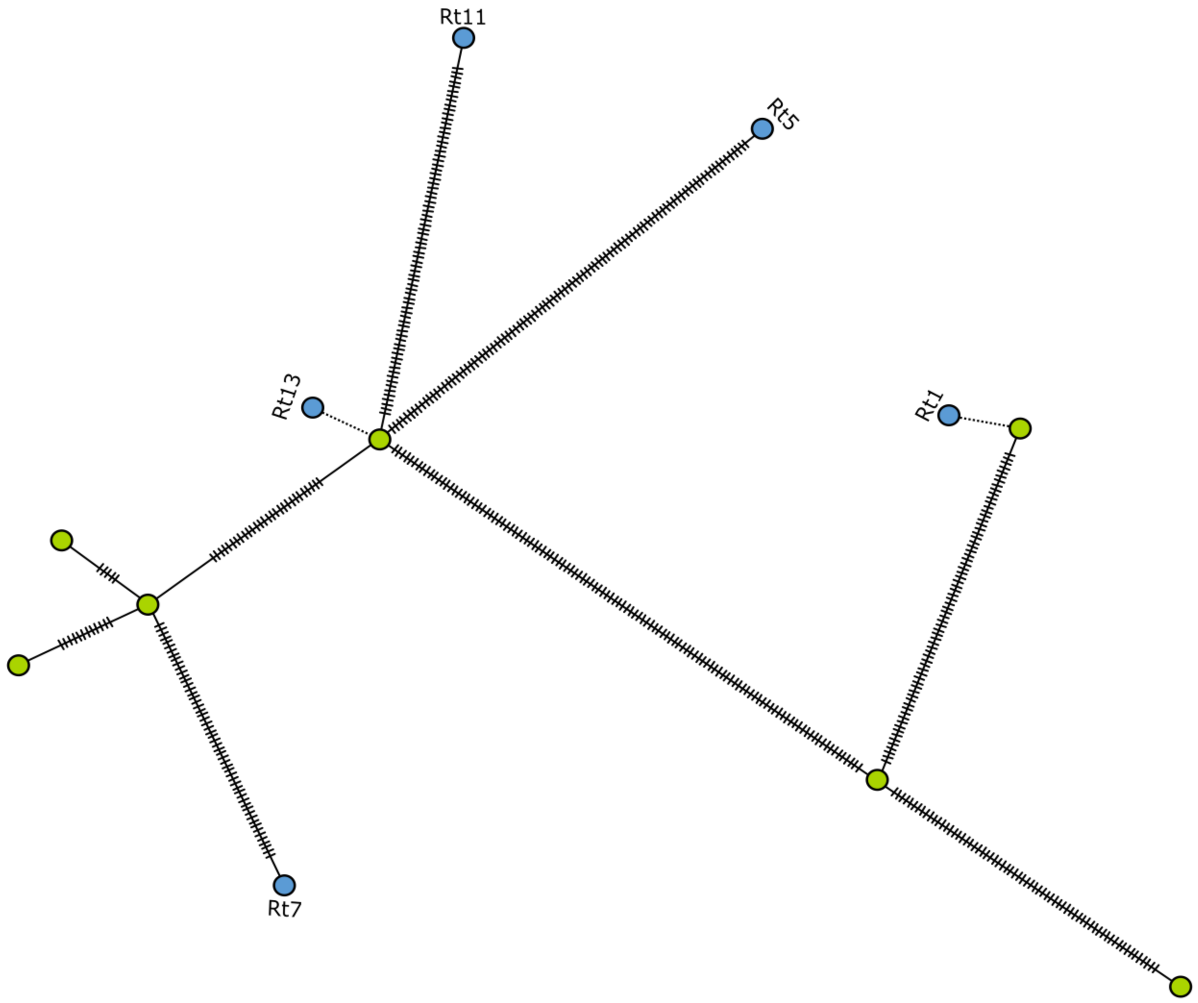

Supplementary Fig. S14. MtDNA haplotype network for reindeer. Each dot represents a sample, with ticks on the connecting lines showing the number of base pair substitutions between the haplotypes. Dotted lines radiating from dental calculus samples (in light blue) represent the predicted most closely related haplotype.

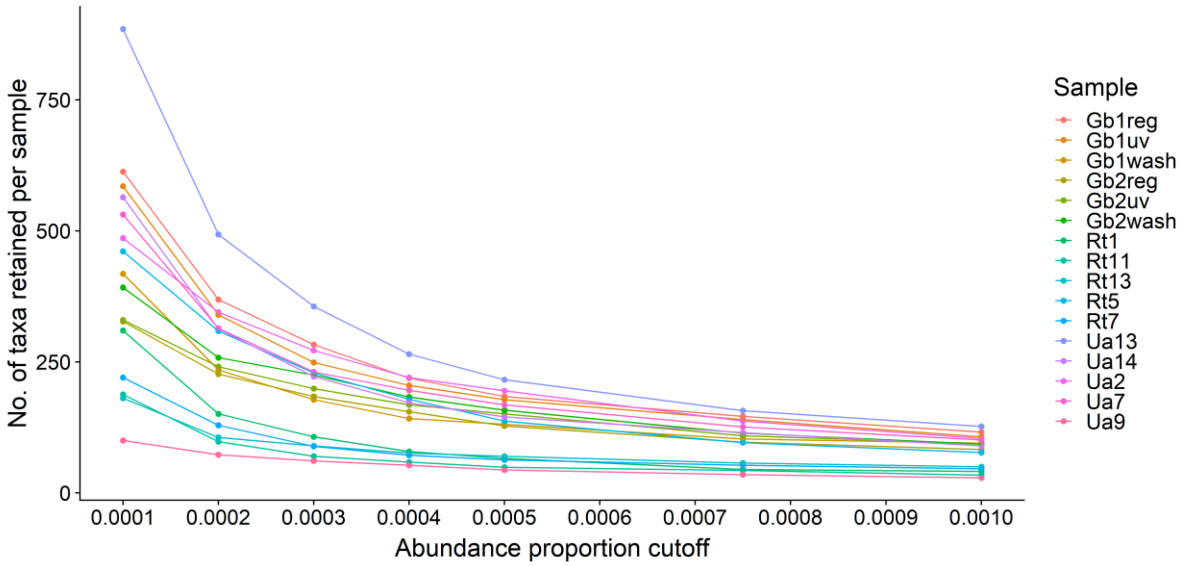

Supplementary Fig. S15. Investigation of abundance filtering thresholds on community complexity, based on microbial relative abundance. The number of taxa retained in each dental calculus sample (coloured lines) at each threshold is shown.

Supplementary Table legends

Supplementary Table S1. Sample metadata.

Supplementary Table S2. PERMANOVA results from microbial distance analyses.

Supplementary Table S3. Oral taxa (at the species level) in dental calculus samples unique to host species.

Supplementary Table S4. Oral bacterial taxa used in the antimicrobial resistance analysis.

Supplementary Table S5. Medium-quality draft metagenome assembled genome (MAG) assembly statistics and taxonomy.

Supplementary Table S6. Investigation into post-mortem deamination and barcoded adapter bias.

Supplementary Table S7. Host DNA recovery statistics from dental calculus samples.

Supplementary Table S8. Sequencing adapter and real-time PCR assay primer sequences.

Supplementary Table S9. Kraken2 taxa table of (1) summaries of raw reads classified and assigned taxonomy at the genus and species level and (2) "processed read counts" (genome size normalised, contaminant- and abundance-filtered) assigned to genus- and species-level taxonomy.

Supplementary Table S10. MEGAN taxa table of MALT raw alignment counts of reads assigned genus- and species-level taxonomy using the NCBI nt reference database.

Supplementary Table S11. Samples used as sources for SourceTracker analysis.

Supplementary Table S12. GenBank accessions of taxa identified and removed as contaminants.
